# Serine-rich repeat protein adhesins from *Lactobacillus reuteri* display strain specific glycosylation profiles

**DOI:** 10.1093/glycob/cwy100

**Published:** 2018-11-23

**Authors:** Dimitrios Latousakis, Ridvan Nepravishta, Martin Rejzek, Udo Wegmann, Gwenaelle Le Gall, Devon Kavanaugh, Ian J Colquhoun, Steven Frese, Donald A MacKenzie, Jens Walter, Jesus Angulo, Robert A Field, Nathalie Juge

**Affiliations:** 1 The Gut Microbes and Health Institute Strategic Programme, Quadram Institute Bioscience, Norwich Research Park, Norwich, UK; 2 School of Pharmacy, University of East Anglia, Norwich Research Park, Norwich, UK; 3 Department of Biological Chemistry, John Innes Centre, Norwich Research Park, Norwich, UK; 4 Evolve Biosystems, Davis, California, USA; 5 Department of Agricultural, Food and Nutritional Science, University of Alberta, Edmonton, AB, Canada; 6 Department of Biological Sciences, University of Alberta, Edmonton, AB, Canada

**Keywords:** accessory secretion system, glycosyltransferase, gut commensal bacteria, *O*-linked glycosylation, sugar nucleotides

## Abstract

*Lactobacillus reuteri* is a gut symbiont inhabiting the gastrointestinal tract of numerous vertebrates. The surface-exposed serine-rich repeat protein (SRRP) is a major adhesin in Gram-positive bacteria. Using lectin and sugar nucleotide profiling of wild-type or *L. reuteri* isogenic mutants, MALDI-ToF-MS, LC–MS and GC–MS analyses of SRRPs, we showed that *L. reuteri* strains 100-23C (from rodent) and ATCC 53608 (from pig) can perform protein *O*-glycosylation and modify SRRP_100-__23_ and SRRP_53608_ with Hex-Glc-GlcNAc and di-GlcNAc moieties, respectively. Furthermore, *in vivo* glycoengineering in *E. coli* led to glycosylation of SRRP_53608_ variants with α-GlcNAc and GlcNAcβ(1→6)GlcNAcα moieties. The glycosyltransferases involved in the modification of these adhesins were identified within the SecA2/Y2 accessory secretion system and their sugar nucleotide preference determined by saturation transfer difference NMR spectroscopy and differential scanning fluorimetry. Together, these findings provide novel insights into the cellular *O*-protein glycosylation pathways of gut commensal bacteria and potential routes for glycoengineering applications.

## Introduction

Although originally believed to be restricted to eukaryotes, protein glycosylation, i.e., the covalent attachment of a carbohydrate moiety to specific protein targets, is emerging as an important feature in bacteria and archaea, revealing an important diversity of glycan structures and pathways within and between microbial species (Schäffer and Messner 2017). To date, protein glycosylation has been widely studied in pathogenic bacteria, where glycoproteins are often essential for virulence and pathogenicity (Eichler and Koomey 2017). However, the nature and function of protein glycosylation in gut commensal bacteria remains largely unexplored (Latousakis and Juge 2018).

*Lactobacillus reuteri* is a Gram-positive bacterial symbiont inhabiting the gastrointestinal (GI) tract of a range of vertebrates (including humans) that displays a remarkable degree of host specialization (Oh et al. 2010; Frese et al. 2011; Frese et al. 2013; Wegmann et al. 2015; Duar et al. 2017). One of the mechanisms mediating specific interaction of *L. reuteri* strains with the host is provided by cell surface proteins that facilitate adherence to epithelial or mucosal surface along the GI tract, depending on the niche colonized by the bacteria (Mackenzie et al. 2010; Etzold et al. 2014; Sequeira et al. 2018). Previous analyses of the rodent strain *L. reuteri* 100-23C identified a gene encoding a predicted surface-exposed serine-rich repeat protein (SRRP_100-23_) that was essential for *L. reuteri* biofilm formation in the forestomach of mice (Frese et al. 2013). Inactivation of SRRP_100-__23_ completely abrogated epithelial association, indicating that initial adhesion represented the most significant step in biofilm formation, likely conferring host specificity (Frese et al. 2013).

SRRPs are a family of adhesins found in many Gram-positive bacteria (Lizcano et al. 2012). These proteins were originally identified in pathogenic bacteria, such as streptococci and staphylococci (Wu et al. 1998; Bensing and Sullam 2002; Zhou and Wu 2009; Seo et al. 2013; Li et al. 2014), where their expression has been linked to virulence (Shivshankar et al. 2009; Sanchez et al. 2010). SRRPs are composed of distinct subdomains: a cleavable and unusually long signal peptide which, in some cases, is followed by an alanine-serine-threonine rich (AST) motif, a short serine rich repeat region (SRR1), a binding region (BR), a second and much larger SRR2, and an LPXTG cell wall anchoring motif (Rigel and Braunstein 2008). Previous studies on SRRPs from pathogenic organisms have shown that these proteins are *O*-glycosylated on serine or threonine residues and exported via an accessory secretion (SecA2/Y2) system (Bensing and Sullam 2002; Bensing et al. 2004; Takamatsu et al. 2004; Siboo et al. 2008; Chaze et al. 2014; Li et al. 2014). This specialized secretion system is encoded by genes that are normally co-located within a gene cluster and is composed of the motor protein SecA2, the translocon channel SecY2 and three to five accessory Sec proteins (Asp1-5). In addition, this gene cluster also contains genes encoding a variable number of glycosyltransferases (GTs), ranging between two and ten (Bensing et al. 2014). The best studied examples of SecA2/SecY2-mediated glycosylation systems are from pathogenic *Streptococcus parasanguinis*, *Streptococcus pneumoniae*, *Streptococcus gordonii*, *Streptococcus agalactiae* and *Staphylococcus aureus* (Takamatsu et al. 2004; Zhu et al. 2016; Jiang et al. 2017). In all cases, the glycosylation process is initiated by a two-protein glycosyltransferase complex, consisting of GtfA and GtfB, that mediate the addition of *N*-acetylglucosamine (GlcNAc) to serine and threonine residues within the SRR domains of the adhesins. This is sometimes followed by the extension of the core glycan via the action of additional GTs whose number and type vary between species, resulting in a range of glycan structures (Zhu et al. 2016; Jiang et al. 2017; Chen et al. 2018). Recently, a SecA2/Y2 cluster encoding three SRRPs has been identified in the commensal species *Streptococcus salivarius* JIM8777; unusually the first glycosylation step was carried out by two genetically linked GTs outside of the cluster (Couvigny et al. 2017).

To date, SecA2/Y2 clusters have been identified in the genomes of various *Lactobacillus* species (Tytgat and de Vos 2016; Latousakis and Juge 2018; Sequeira et al. 2018). In *L. reuteri*, the intact cluster has mostly been found in strains of murine or porcine origin, and it appears to be absent from strains of human origin (Frese et al. 2011; Frese et al. 2013; Wegmann et al. 2015; Sequeira et al. 2018). The SecA2/Y2 cluster in the *L. reuteri* rodent strain 100-23C is crucial for ecological fitness and adhesion of the bacteria to the forestomach epithelium of the murine GI tract (Frese et al. 2013). Using proteomics, we showed that SRRP_100-__23_ is the primary cell wall-associated protein of *L. reuteri* 100-23C strain that is secreted through the accessory SecA2/Y2 system (Frese et al. 2013). In addition, our analysis of the completed genome of the pig isolate *L. reuteri* ATCC 53608 revealed the presence of a SecA2/Y2 system with an associated SRRP sharing the same domain organization as SRRP_100-__23_ (Wegmann et al. 2015). Further analysis of the pangenome of *L. reuteri* pig isolates also revealed the presence of a SecA2/Y2 system with an associated SRRP in these strains (Wegmann et al. 2015), suggesting a conserved role of SecA2/Y2 among *L. reuteri* strains that possess the cluster. We confirmed that the SRRPs from *L. reuteri* pig strains were secreted during growth *in vitro* (Sequeira et al. 2018), as previously shown for SRRP_100-__23_ (Frese et al. 2013). However, despite the central importance of the SecA2/Y2 cluster and SRRPs in specific *L. reuteri* strains, how SRRPs are glycosylated in lactobacilli has not yet been determined.

Here we provide a comprehensive analysis of the glycosylation of *L. reuteri* SRRPs (*Lr*SRRPs) from *L. reuteri* ATCC 53608 (pig) and 100-23C (rodent) strains. Using a combination of bioinformatics analysis, lectin screening, LC–MS-based sugar nucleotide profiling, MALDI-ToF and GC–MS analyses, we showed that the *L. reuteri* ATCC 53608 and 100-23C strains are capable of performing protein glycosylation and that SRRP_100-__23_ and SRRP_53608_ are glycosylated with hexose (Hex)_2_-*N*-acetylhexosamine (HexNAc) and di-HexNAc moieties, respectively. Following *in vivo* glycoengineering in *E. coli*, NMR analysis and enzymatic treatment showed that SRRP_53608_ is glycosylated with GlcNAcβ(1→6)GlcNAcα moieties. In addition, using Differential Scanning Fluorimetry (DSF) and Saturation Transfer Difference (STD) NMR, we provide biochemical insights into the specificity of the glycosyltransferases involved in the SecA2/Y2 accessory pathway leading to the protein glycosylation of these adhesins in gut symbionts.

## Results

### SRRPs from *L. reuteri* strains 100-23C and ATCC 53608 are glycosylated

**Fig. 1.**
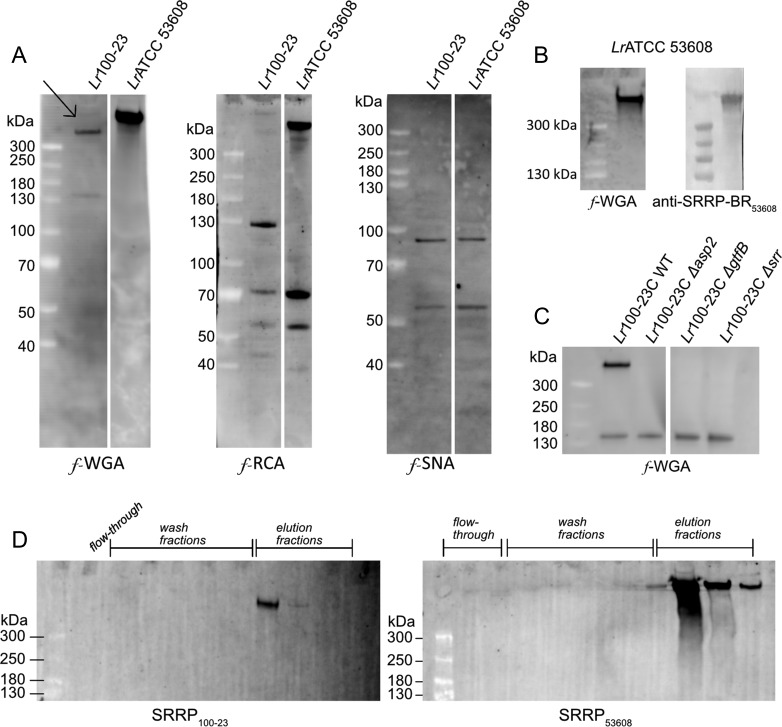
Lectin screening of *L. reuteri* SM proteins. (**A**) Western blot analysis of *L. reuteri* 100-23C and ATCC 53608 SM proteins, using *f*-WGA, *f*-RCA and *f*-SNA. The arrow indicates SRRP in *L. reuteri* 100-23C. (**B**) Western blot analysis of *L. reuteri* ATCC 53608 SM proteins with *f*-WGA and anti-SRRP-BR_53608_ antibody. (**C**) Western blot analysis of *L. reuteri* 100-23C WT, *Δasp2, ΔgtfB* and *Δsrr* mutant SM proteins with *f*-WGA. (**D**) Purification of *Lr*SRRPs by affinity chromatography, using agWGA. *Lr*SRRPs were eluted with 0.5 M GlcNAc.

To determine whether *L. reuteri* strains 100-23C and ATCC 53608 are capable of performing protein glycosylation of *Lr*SRRPs, the proteins from the spent media (SM) were separated by SDS-PAGE and analyzed by western blot using a range of fluorescein (*f*)-labeled lectins. A similar lectin recognition profile was observed between proteins from both *L. reuteri* strains with binding to *f-*WGA, *f-*RCA and *f-*SNA (Figure 1A) while no binding was observed with *f-*ConA, *f-*LTL, *f-*PNA or *f-*UEA (data not shown). This suggests the presence of glycoproteins carrying GlcNAc, sialic acid or galactose (Gal) residues. A large protein with an apparent molecular weight (MW) > 300 kDa was detected in both *L. reuteri* strains by *f-*WGA but not with any of the other lectins tested. This protein was also recognized by anti-SRRP-BR_53608_ antibodies in *L. reuteri* ATCC 53608 SM, suggesting that it corresponds to SRRP_53608_ (Figure 1B). It is of note that Coomassie-staining cannot efficiently detect *Lr*SRRPs, probably due to their unusual amino acid composition and glycosylation. The anti-SRRP-BR_53608_ does not cross-react with SRRP_100-__23_ which may be due to the low amino acid similarity (48%) between the two binding regions of the two adhesins (Sequeira et al. 2018). Previous reports have also shown that lectins can detect SRRPs with greater sensitivity than antibodies, since the high degree of glycosylation masks the underlying amino acid and protein antigens (Siboo et al. 2008). Therefore, to confirm the identity of the putative SRRP glycoprotein secreted by *L. reuteri* 100-23C, the lectin binding profile of *L. reuteri* 100-23C *Δsrr* mutant (lacking SRRP_100-23_ expression, see (Frese et al. 2013)) was determined as above following western blot analysis with *f*-labeled lectins. The protein band >300 kDa recognized by *f-*WGA in the *L. reuteri* 100-23C wild-type strain was missing in the *Δsrr* mutant (Figure 1**C**) while no other difference in the lectin recognition pattern was observed with *f*-WGA or when the SM proteins were probed with *f*-RCA or *f*-SNA (data not shown), confirming that this protein is SRRP_100-23_ (marked with an arrow in Figure 1A). It is interesting to note that the theoretical MW of SRRP_53608_ and SRRP_100-23_ is 116 kDa and 224 kDa, respectively, therefore the high apparent MW of *Lr*SRRPs is in line with the potential glycosylation of these adhesins. The lectin recognition pattern of *Lr*SRRPs suggests that these adhesins are glycosylated with glycans carrying GlcNAc residues.

**Fig. 2.**
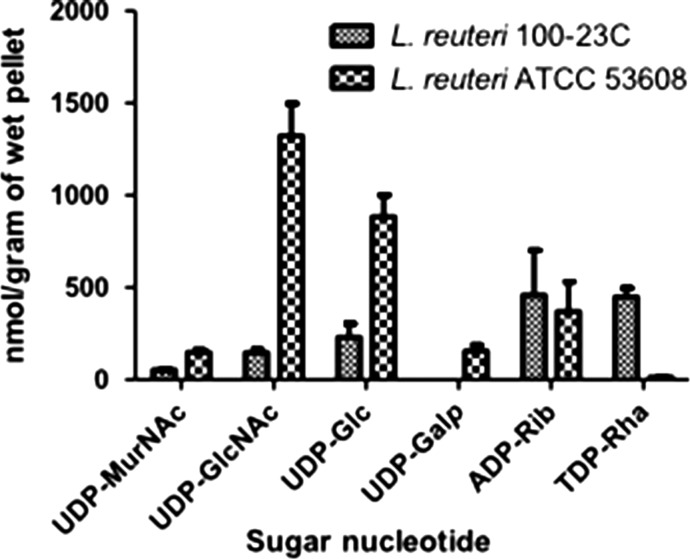
LC–MS sugar nucleotide profiling of *L. reuteri* 100-23C and ATCC 53608 strains. The bars represent the standard error of three biological replicates. See also Table S1 for MRM transitions, retention times and quantity of the sugar nucleotides.

In support of this analysis, the profile of intracellular sugar nucleotides produced by *L. reuteri* strains was determined as described in Rejzek et al. (2017) with some modifications specific for the cell lysis of Gram-positive bacteria. The LC–MS/MS based analysis revealed the presence of six abundant nucleotide 5’-diphosphosugar (NDP-sugar) species in *L. reuteri* 100-23C and ATCC 53608 (Figure 2) at concentrations ranging from low nmol to low μmol per gram of wet cell pellet (Table S1). UDP-GlcNAc and UDP-Glc were detected in both *L. reuteri* strains at high levels (Figure 2). UDP-Gal was also found in both strains but at significantly lower levels in *L. reuteri* 100-23C, under the conditions tested. These results are in line with the bioinformatics analyses showing the genetic requirement for the synthesis of UDP-GlcNAc, UDP-Glc, UDP-Gal (data not shown) which are commonly used as sugar donors by GTs in protein glycosylation (Freeze et al. 2017) and in agreement with the presence of GlcNAc moieties onto *Lr*SRRPs, as suggested by the lectin screening.

### SRRP_100-23_ and SRRP_53608_ are glycosylated with Hex_*2*_GlcNAc and di-GlcNAc moieties, respectively

**Fig. 3.**
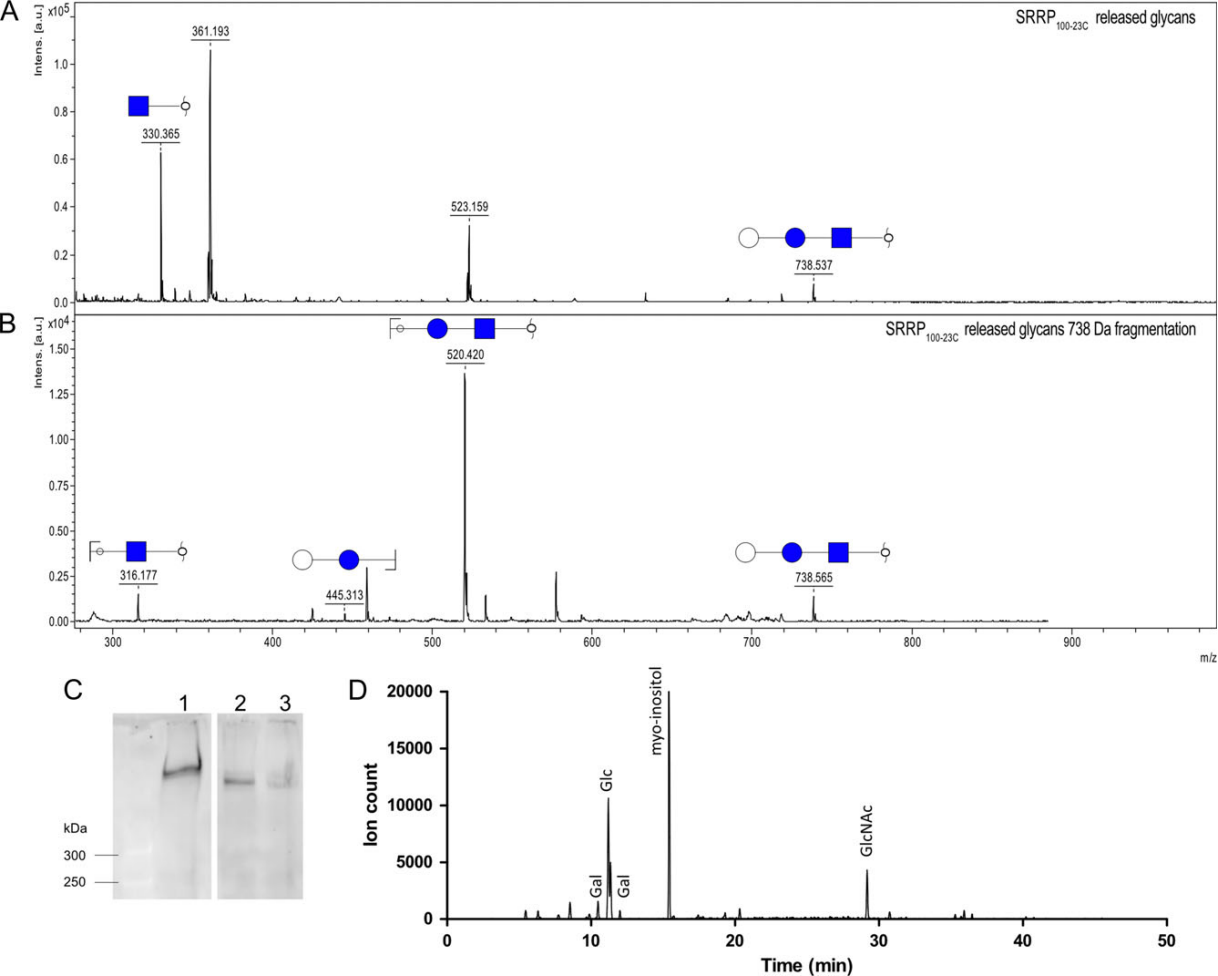
Structural analysis of SRRP_100-23_ glycosylation. (**A**) MALDI-ToF analysis of SRRP_100-23_ released glycans found in the 35% ACN elution fraction. (**B**) Fragmentation of the 738 Da peak. (**C)** Western blot analysis of enzymatically deglycosylated SRRP_100-23_. 1. SRRP_100-23_ (1), treated with α- and β-glucosidase (2), or α- and β- galactosidase (3). (**D**) Monosaccharide composition analysis of SRRP_100-23_ glycans. Extracted ion chromatogram for ions at 204 and 173 Da, characteristic for monosaccharides. See also Figure S1 for comparison of MALDI-ToF spectra of the fraction containing the released glycans of *L. reuteri* 100-23 WT and *ΔgtfB* mutant.

To identify the glycans decorating *Lr*SRRPs, SRRP_100-23_ and SRRP_53608_ were purified from *L. reuteri* culture supernatant by affinity chromatography using an agarose-bound WGA (agWGA) column. The purified proteins migrated at a MW > 300 kDa on SDS-PAGE and were recognized by *f*-WGA (Figure 1D) on western blot. The purified *Lr*SRRPs were then subjected to reductive β-elimination, and the chemically released glycans permethylated and analyzed by MALDI-ToF. The spectra of SRRP_100-23_ showed a peak at 738 Da, corresponding to Hex_2_HexNAc (Figure 3A) and fragmentation of this ion species suggested a linear glycan structure (Figure 3B). The peak at 330 Da corresponds to reduced, permethylated HexNAc, suggesting some degree of heterogeneity in the glycosylation of SRRP_100-23_ which may also explain the recognition of SRRP_100-23_ by WGA. Interestingly, the Hex-HexNAc intermediate could not be identified in the sample. As further support of SRRP_100-23_ glycosylation, SM proteins from *L. reuteri* 100-23C *asp2* and *gtfB* mutants (Frese et al. 2013) were analyzed by western blot using *f-*WGA. The WGA-band corresponding to SRRP_100-23_ was missing in both mutants (Figure 1C) and glycomics analysis of SM proteins from the *gtfB* mutant showed a loss of the peak at 738 Da compared to the wild-type strain (Supplementary data, Figure S1), further confirming that this modification was due to SecA2/Y2-mediated protein glycosylation. To identify the nature of the monosaccharides constituting SRRP_100-23_ glycans, the adhesin was treated with α- or β-glucosidase, or α-, or β-galactosidase and the reaction product was analyzed by western blot, using *f*-WGA. The results showed that treatment with either α-glucosidase or α-galactosidase led to reduction of the apparent MW of the adhesin after SDS-PAGE (Figure 3C), suggesting that the terminal hexoses could be either Glc or Gal. Further analysis of the monosaccharides in the elution fraction of the agWGA affinity chromatography by GC–MS, following methanolysis, *N*-acetylation and TMS-derivatization of the released methyl-glycosides, showed that Glc and Gal were the only hexoses present, supporting the enzymatic deglycosylation data (Figure 3D). The analysis also showed that GlcNAc was the only HexNAc present. Together these results suggest that SRRP_100-23_ is modified with GlcNAc and Glc or Gal moieties with GlcNAc being at the reducing end of the glycans.

**Fig. 4.**
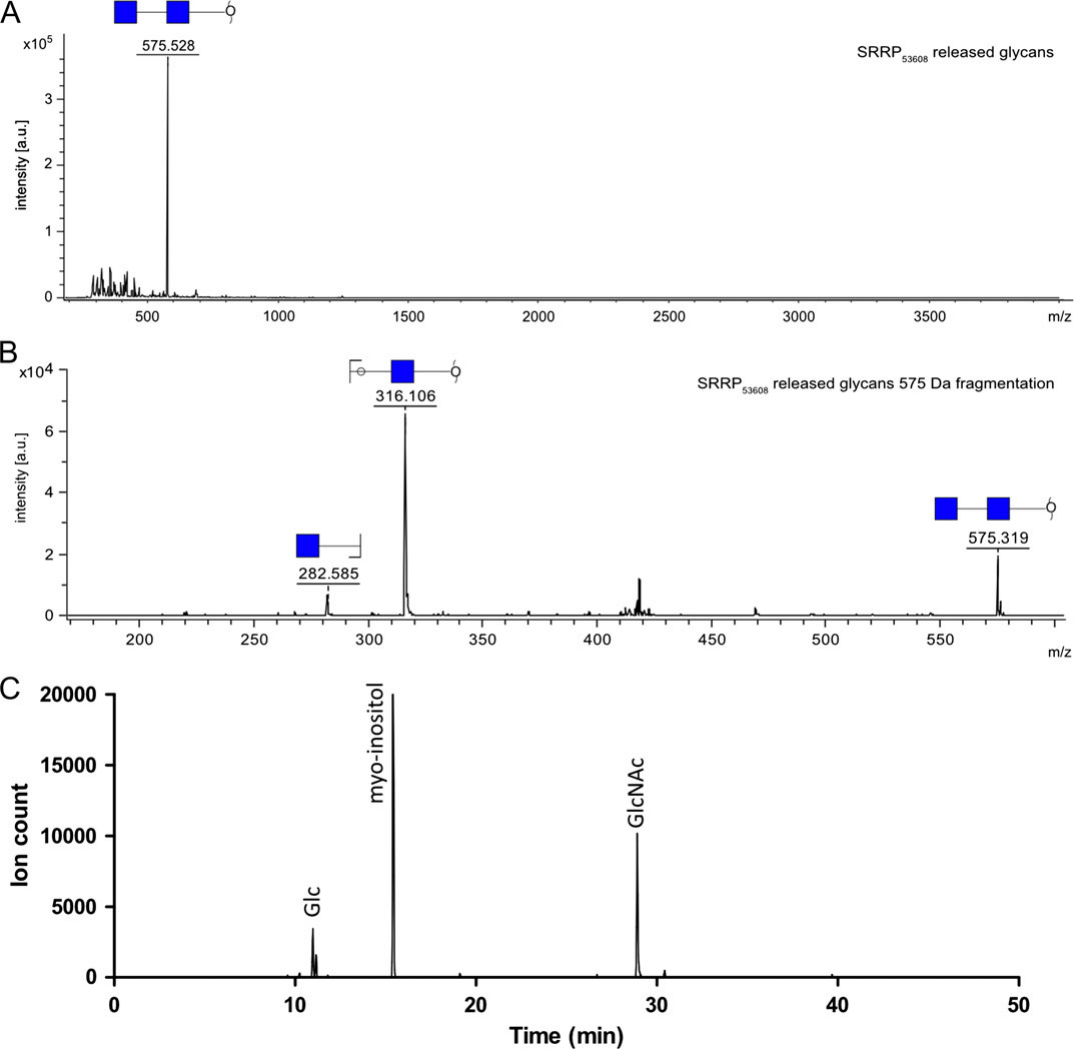
Structural analysis of SRRP_53608_ glycosylation (**A**) MALDI-ToF analysis of SRRP_53608_ released glycans. (**B**) Fragmentation of the 575 Da peak. (**C**) Monosaccharide composition analysis of SRRP_53608_ glycans. Extracted ion chromatogram for ions at 204 and 173 Da, characteristic for monosaccharides.

MALDI-ToF analysis of SRPP_53608_ glycans revealed a single peak at 575 Da, which corresponds to the mass of a reduced, permethylated sodiated di-HexNAc (Figure 4A). Further fragmentation of this species confirmed the nature of the glycan, as it produced two main peaks at 282 Da and 316 Da, corresponding to a non-reducing and a reducing terminal HexNAc, respectively (Figure 4B). To determine the nature of the glycan residues, the carbohydrate content of purified SRRP_53608_ was further analyzed by GC–MS. The chromatogram showed a single HexNAc peak with a retention time (~29 min) corresponding to that of GlcNAc (Figure 4C).

Taken together, these data suggest that SRRP_100-23_ is mainly glycosylated with Hex-Hex-GlcNAc and SRRP_53608_ with di-GlcNAc moieties. These results are in agreement with the lectin and sugar nucleotide profiling of *L. reuteri* strains 100-23C and ATCC 53608.

### SRRP_100-23_ and SRRP_53608_ display different glycosylation pathways

**Fig. 5.**
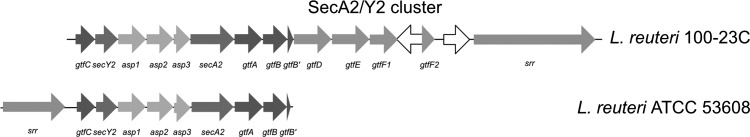
Schematic representation of the accessory SecA2/Y2 clusters from *L. reuteri* 100-23C and ATCC 53608.

**Fig. 6.**
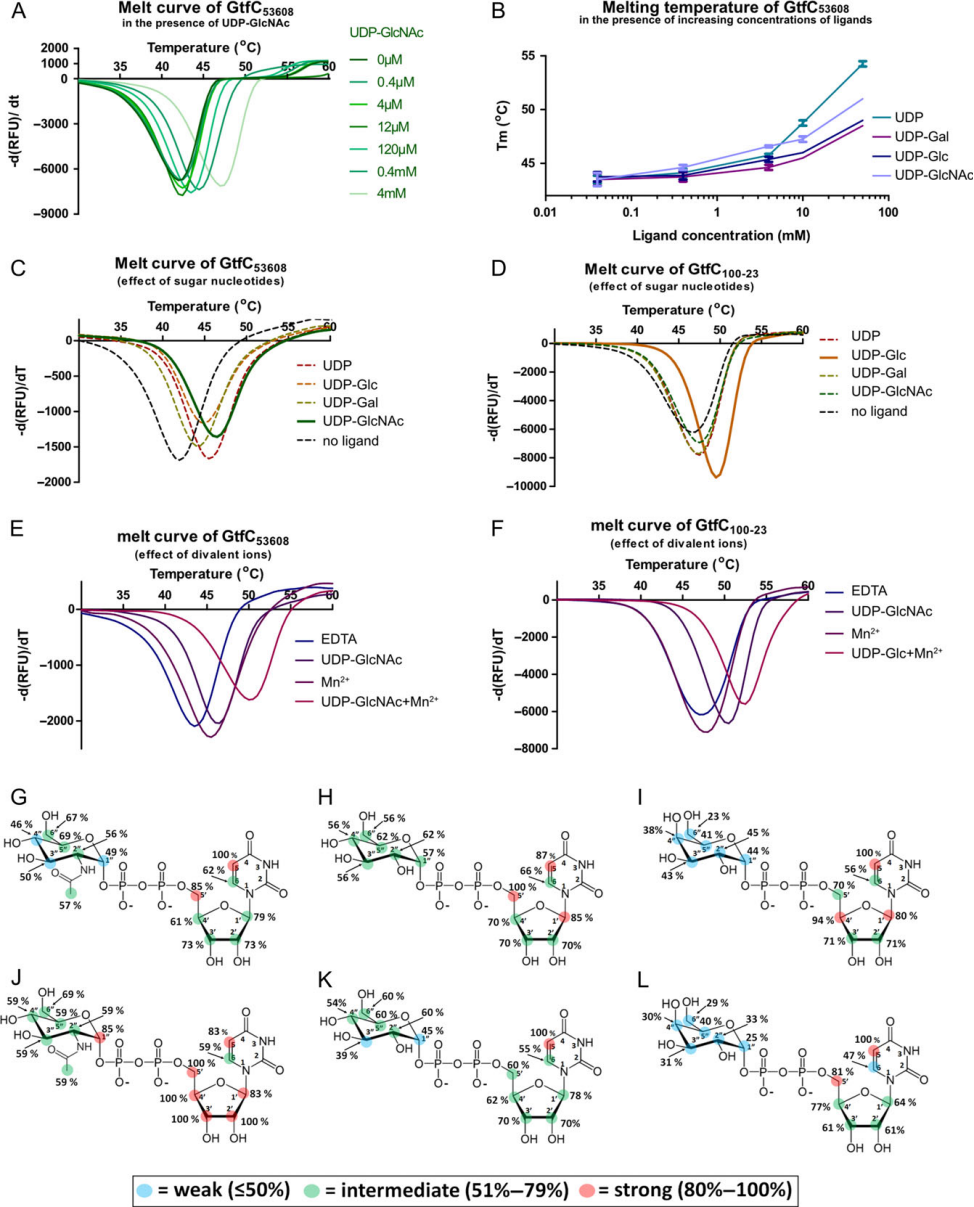
Analysis of GtfC_100-23_ and GtfC_53608_ ligand specificity. (**A–F**) Differential scanning fluorimetry (DSF) analysis. (**A**) Melt curve of GtfC_53608_ in the presence of increasing concentrations of UDP-GlcNAc. (**B**) Tm of GtfC_53608_ in the presence of increasing concentrations of UDP, UDP-Gal, UDP-Glc and UDP-GlcNAc. Error bars represent the standard error of the mean of four technical replicates. (**C**) Melt curve of GtfC_53608_ in the presence of 4 mM UDP-GlcNAc, UDP-Glc, UDP-Gal and UDP. (**D**) Melt curve of GtfC_100-23C_ in the presence of 4 mM UDP-GlcNAc, UDP-Glc, UDP-Gal and UDP. (**E**) Melt curves of GtfC_53608_ in the presence of 5 mM Mn^2+^ (left), or 5 mM Mn^2+^ and 4 mM UDP-GlcNAc. (**F**) Melt curves of GtfC_100-23C_ in the presence of 5 mM Mn^2+^ (left), or 5 mM Mn^2+^ and 4 mM UDP-Glc. Since no significant difference was observed between the different divalent ions, only Mn^2+^ is shown. (**G–L**) Saturation Transfer Difference (STD) NMR analysis. (**G**), (**H**), (**I**) Binding epitope maps for the complexes of GtfC_100-23_ with UDP-GlcNAc, UDP-Glc and UDP-Gal, respectively. Bottom row, (**J**), (**K**), (**L**) binding epitope maps for the complexes of GtfC_53608_ with UDP-GlcNAc, UDP-Glc and UDP-Gal, respectively. See also Table I and Figure S2 for the competition assays of the sugar nucleotides against GtfC_100-23_ and GtfC_53608_.

In addition to the SecA2 and SecY2 translocases and the accessory secretion associated proteins Asp1-3, the *L. reuteri* ATCC 53608 SecA2/Y2 glycosylation system contains genes encoding the priming GtfA_53608_ and GtfB_53608_, and a gene encoding GtfC_53608_ (Figure 5) whereas, in *L. reuteri* 100-23C, the SecA2/Y2 cluster includes eight genes encoding predicted GTs, including GtfA_100-23_, GtfB_100-23_ and GtfC_100-23_ (Figure 5). Based on homologous SecA2/Y2 clusters in streptococcal and staphylococcal systems, GtfA and GtfB are predicted to act together to initiate glycosylation of SRRPs by the addition of a GlcNAc residue, whereas GtfC is predicted to mediate the second glycosylation step (Zhu et al. 2016; Couvigny et al. 2017; Jiang et al. 2017). Based on the SRRP_100-23_ and SRRP_53608_ glycosylation profiles determined above, GtfC_53608_ and GtfC_100-23_ are predicted to add a GlcNAc residue or a Hex residue, respectively, to the GlcNAc core, while sharing 97% identity in amino acid sequence (Supplementary data, Figure S2). To confirm the ligand specificity of these enzymes, GtfC_53608_ and GtfC_100-23_ were heterologously expressed in *E. coli* and the recombinant enzymes first analyzed by differential scanning fluorimetry (DSF). Interactions of proteins with their ligands often lead to increased stabilization of the protein, and this is reflected by an increased melting temperature (Tm) (D’Urzo et al. 2012). GtfC_53608_ showed a UDP-GlcNAc concentration-dependent increase in Tm, from 42°C in the absence of the ligand to 47°C in the presence of 4 mM UDP-GlcNAc (Figure 6A). The specificity of GtfC_53608_ interaction was further tested against UDP, UDP-Gal and UDP-Glc, showing a concentration-dependent increase in Tm for all ligands tested (Figure 6B) but lower than the interaction with UDP-GlcNAc (Figure 6B, C), indicating a preference of GtfC_53608_ towards UDP-GlcNAc. GtfC_100-23_ showed an increase in Tm of up to 3°C in the presence of UDP-Glc, whereas other ligands had a reduced effect at concentrations up to 4 mM (Figure 6D), indicating a preference of GtfC_100-23_ for UDP-Glc. DSF was also used to investigate the dependency of GtfC_53608_ and GtfC_100-23_ to metal ions. The Tm of GtfC_53608_ was increased by 2.5°C in the presence of 5 mM of the divalent ions (Mg^2+^, Mn^2+^, Ca^2+^) and by 7°C when both the sugar ligand UDP-GlcNAc and metal ions were present (Figure 6E). A smaller shift in Tm (<1°C) was detected when the ions were added to GtfC_100-23_ in the absence or presence of UDP-Glc (Figure 6F). These results suggest that GtfC_53608_ and GtfC_100-23_ have different requirements for divalent ions for optimum binding.

Saturation Transfer Difference (STD) NMR was used to obtain structural insights into the interaction between GtfC_53608_ or GtfC_100-23_ and these sugar nucleotides. We obtained binding epitope maps (maps of distribution of STD_0_(%) factors along the molecule) for each ligand tested (UDP, UDP-Gal, UDP-Glc and UDP-GlcNAc), reflecting the main contacts with the surface of the protein in the bound state. For each ligand, the highest STD_0_(%) factors were observed for the uracil and ribose moieties whereas the hexopyranose moieties (Glc, GlcNAc and Gal) showed lower STD_0_(%) factors (Figure 6G–L). In addition, there were differences between the ligand binding epitopes in complex with GtfC_53608_ or GtfC_100-23_. UDP-GlcNAc showed higher STD_0_(%) factors on average in the presence of GtfC_53608_ (Figure 6J), supporting a preference of this protein for UDP-GlcNAc whereas GtfC_100-23_ showed a binding preference for UDP-Glc (Figure 6H). UDP-Gal showed only weak interactions with GtfC_100-23_ or GtfC_53608_ (Figure 6I, L). STD NMR titrations were carried out to determine the ligand affinity of GtfC_53608_ and GtfC_100-23_. Since the stability of the protein samples imposed time constraints on the NMR measurements precluding an STD initial slope titration approach to get thermodynamic values (Angulo et al. 2010), the *K*_D_ values were considered as apparent. All apparent *K*_D_ values, were in excellent agreement with the binding epitope data, except for the *K*_D_ of the complex GtfC_100-23_/UDP-Gal which was lower than GtfC_100-23_/UDP-Glc. In order to explore this further, a competitive STD NMR study was performed where the STD factors for the complexes GtfC_100-23_/UDP-Glc, GtfC_100-23_/UDP-GlcNAc, GtfC_53608_/UDP-GlcNAc and GtfC_53608_/UDP-Glc were determined in the absence or presence of UDP-Gal. The results (Table I, Supplementary data, Figure S3) were in excellent agreement with the epitope mappings of the sugar nucleotides, supporting the preference of GtfC_100-23_ towards UDP-Glc, despite the lower apparent *K*_D_ obtained for UDP-Gal. The difference in apparent *K*_D_ may be due to a conformational rearrangement of GtfC_100-23_ in the presence of UDP-Glc, reducing the kinetics rate of the association process (on-rate, k_ON_), leading to an underestimation of affinity due to ligand rebinding (Angulo et al. 2010), as was previously reported for the complex of the human blood group B galactosyltransferase and its donor substrate UDP-Gal (Angulo et al. 2006).

**Table I.** Affinity ranking of UDP, UDP-GlcNAc, UDP-Glc and UDP-Gal for GtfC_53608_ and GtfC_100-23_ from different ^1^H STD NMR approaches

STD-NMR determination of the ligand affinity of GtfC_100-23_ and GtfC_53608_				
Ligands	GtfC_53608_		GtfC_100-23_	
	*K*_D_ (mM)	Affinity from competition	*K*_D_ (mM)	Affinity from competition
UDP-Glc	1.8	+	0.99	++++
UDP-GlcNAc	0.43	++++	2.4	+
UDP-Gal	1.66	+	0.31	+

Taken together, these results suggest that GtfA/B are involved in GlcNAc attachment to SRRP_100-23_ and SRRP_53608_ while GtfC_53608_ extends the chain with a GlcNAc residue and GtfC_100-23_ with Glc.

### *In vivo* glycoengineering of SRR1 domain

To gain further insights into the glycosylation of SRRP_53608_, a sequence encoding a His-tagged SRR1 region covering aa 81-236 of SRRP_53608_ was co-expressed in *E. coli* together with an operon encoding GtfA_53608_, GtfB_53608_ and GtfC_53608_. MS analysis after trypsin digest of protein bands at 60, 50 and 40 kDa (Supplementary data, Figure S4A), confirmed that these correspond to the successfully expressed GtfA_53608_, GtfB_53608_ and GtfC_53608_, respectively (data not shown). The protein extract was further analyzed by western blot, using *f*-WGA. A protein migrating between 45 and 60 kDa was detected by *f*-WGA when GtfA/B/C_53608_ and SRR1, were co-expressed, but not in the control experiment expressing SRR1 only (Supplementary data, Figure S4B), suggesting that this protein corresponds to glycosylated SRR1 (gSRR1). The his-tagged gSRR1 was purified by IMAC and subjected to reductive β-elimination. Analysis of the permethylated glycans by MALDI-ToF MS showed a peak at 575 Da (Supplementary data, Figure S5A), consistent with the presence of di-HexNAc species, as seen for the glycans from the native SRRP_53608_. The assignment of this peak as a di-HexNAc-ol was also supported by fragmentation of the species at 575 Da that showed dominant peaks at 316 and 282 Da (Supplementary data, Figure S5A). Two weak signals at 330 Da and at 534 Da, corresponding to the mass of a permethylated, sodiated HexNAc and Hex-HexNAc-ol, respectively, were also observed (Supplementary data, Figure S5A).

The released, underivatized glycans were analyzed using 2D NMR and DEPT experiments in order to characterize the conformation and linkage of the disaccharide. NMR spectra of α/ β-GlcNAc and GlcNAc-ol standards were recorded for comparison with the experimental samples. The NMR analysis of the gSRR1 glycans confirmed the presence of a di-GlcNAc disaccharide (Table II), in agreement with the MS analysis of gSRR1 and the glycosylation of native SRRP_53608_. The disaccharide was determined to be β-GlcNAc-(1→6)-GlcNAc-ol (Supplementary data, Figure S5B-C). In addition, the released glycan fraction also revealed the presence of free GlcNAc-ol and the two mixture components were present in the proportions GlcNAc-ol (60%): disaccharide (40%) (Supplementary data, Figure S5B), suggesting that the glycosylation of gSRR1 in *E. coli* consists of a combination of mono- and di-GlcNAc side chains. A minor doublet was detected at 4.50 ppm, suggesting the presence of a second disaccharide on gSRR1, in agreement with the MALDI-ToF analysis that showed the presence of a Hex-HexNAc-ol. The β-conformation of the non-reducing GlcNAc was further confirmed by treatment of recombinant gSRR1 with a commercially available β-*N*-acetylhexosaminidase_*f*_. The enzymatically-treated gSRR1 showed reduced apparent size on western blot following detection by *f*-WGA as compared to non-treated gSRR1 (Supplementary data, Figure S5C).

**Table II.** ^1^H and ^13^C chemical shifts of reference standards, glycan released from gSRR1 and glycan units present in intact gSRR1

NMR characterization of the sSRR1 released glycans									
		1	2	3	4	5	6	CH3	C=O
Reference Standards									
α-GlcNAc	H	5.21	3.88	3.78	3.50	3.86	3.86,3.80	2.06	–
	C	93.70	56.96	73.52	72.91	74.44	63.42	24.77	177.40
β-GlcNAc	H	4.72	3.68	3.55	3.47	3.47	3.92,3.76	2.06	–
	C	97.79	59.54	76.73	72.69	78.81	63.58	25.05	177.65
GlcNAc-ol (R)	H	3.64,3.74	4.08	3.97	3.60	3.76	3.66,3.83	2.06	–
	C	63.68	56.58	71.14	73.79	73.93	65.62	24.96	177.35
Glycan released from gSRR1, β-GlcNAc-(1→6)-GlcNAc-ol									
β-GlcNAc(1→(B)	H	4.55	3.75	3.57	3.46	3.47	3.95,3.76	2.07	–
	C	104.45	58.44	76.65	72.81	78.68	63.58	25.09	177.65
→6)GlcNAc-ol (G)	H	3.64,3.74	4.08	3.97	3.60	3.84	4.09	2.05	–
	C	63.73	56.55	70.95	73.65	72.49	73.75	24.94	177.35
GlcNAc units present in gSRR1, M = monosaccharide, D = disaccharide side-chain									
t-α-GlcNAc→Ser (αM)	H	4.87	3.92	3.72	3.47	3.62	3.84,3.78	~2.05	–
	C	100.61	56.35	73.86	72.68	75.15	63.44	~25.0	~177.0
→6)-α-GlcNAc→Ser (αD)	H	4.88	n.d.	n.d	n.d.	n.d.	4.13,3.80	n.d.	n.d.
	C	100.61	n.d.	73.87	72.54	n.d.	71.13	n.d.	–
t-β-GlcNAc(1→(βD)	H	4.54	3.75	3.58	3.47	3.47	3.94,3.77	~2.07	–
	C	104.51	58.41	76.54	72.67	78.74	63.68	~25.2	~177.3

See also Supplementary data, Figure S5 and Table S3 for information on the expression of GtfA, GtfB and GtfC, and glycosylation of gSRR1 and Supplementary data, Figures S5 and S6 for information on the structural characterization of the gSRR1 released and native glycans by NMR.

n.d. = not determined.

To determine the configuration of GlcNAc linked to the protein, NMR experiments were carried out on the intact gSRR-1 protein. NMR assignments of the sugar residues in gSRR1 are reported in Table II and details of how the assignments were made are provided in the Supplementary data, Figure S5 captions (Supplementary data, Figure S6). The analysis revealed that GlcNAc was α-linked to gSRR1 and confirmed that both single α-GlcNAc and GlcNAcβ-(1→6)-GlcNAcα disaccharide side chains were present. In the ^1^H spectrum of gSRR1, the anomeric signal of β-GlcNAc appeared as a simple doublet, *J*_1,2_ = 8.6 Hz, at δ 4.54, but the anomeric signal of α-GlcNAc appeared as a broad feature centered at δ 4.87. This broad feature consisted of a superposed series of doublets, all with *J*_1,2_ = 3.9 Hz, but with displaced δH1 chemical shifts in the range 4.91–4.85 ppm (Supplementary data, Figure S6C). The displacement arises because the sugars are linked to serine or threonine residues that occupy slightly different environments as a result of the protein secondary structure. By integrating the α- and β- ^1^H anomeric signals (Supplementary data, Figure S6D) it was possible to estimate the proportions of mono- to disaccharide side chains as 64%:36%, in agreement with the result obtained from the released glycans mixture.

Together these data showed that GtfA, GtfB and GtfC can glycosylate gSRR1 in *E. coli*. Detailed NMR analysis of the intact glycoprotein, as well as the released glycans, showed that gSRR1 is modified with α-linked GlcNAc residues and GlcNAcβ(1→6)GlcNAcα moieties at a ~4:6 ratio with a small fraction of a Hex-GlcNAc species further identified by MS and NMR.

## Discussion

Protein glycosylation is emerging as an important feature in bacteria. Protein glycosylation systems have been reported and studied in many pathogenic bacteria, revealing an important diversity of glycan structures and pathways within and between bacterial species. Studies focused on SRRPs from streptococci and staphylococci have demonstrated that these adhesins are *O-*glycosylated. In these closely related bacteria, glycosylation of SRRPs is initiated by a complex between GtfA and GtfB that adds GlcNAc to the SRR domains of the adhesins while additional GTs, including GtfC, may further modify SRR glycosylation by sequentially adding other glycan moieties onto the GlcNAc core (Takamatsu et al. 2004; Shi et al. 2014; Zhu et al. 2016; Jiang et al. 2017). Here we showed that the gut symbiont *L. reuteri* is capable of performing *O*-glycosylation on proteins, and that *L. reuteri* strains differentially modify SRRPs. SRRP_100-23_ is glycosylated with GlcNAc and Hex-Glc-GlcNAc whereas SRRP_53608_ is glycosylated with GlcNAc and di-GlcNAc moieties. *L. reuteri* GtfAB are expected to be involved in the addition of the core GlcNAc to serine or threonine, in agreement with the glycan structure of SRRP_100-23_ and SRRP_53608_ and with their high sequence homology with other functionally characterized GtfAs (e.g., ~46% identity with GtfA from *S. pneumoniae* TIGR4 (Jiang et al. 2017), *E*-value < 10^–150^). In addition to the SecA2/SecY2 export system dedicated to the glycosylation of SRRPs, a general *O*-glycosylation system has been reported in *L. plantarum* WCFS1 where homologs of *L. reuteri* SecA2/Y2 GtfA and GtfB have been shown to be involved in the addition of a single HexNAc molecule onto the glycosylation site of the acceptor proteins (Lee et al. 2014). These two enzymes contain a DUF1975 in the N-terminus which probably mediates the interaction between the two GTs and the target proteins and a GT domain in the C-terminus, as demonstrated for GtfA and GtfB from *S. parasanguinis* FW213 (Wu and Wu 2011), suggesting a similar mode of action to the SecA2/Y2-specific GtfA and GtfB.

The glycosylation of SRRP_100-23_ with Hex-Glc-GlcNAc is in line with the specificity of GtfC_100-23_ to UDP-Glc by DSF and STD NMR. The second Hex (either Glc or Gal) may be the result of another GT present in the *L. reuteri* 100-23C SecA2/Y2 cluster (see Figure 5). The number of GTs in the *L. reuteri* 100-23C SecA2/Y2 cluster exceeds the number of sugars on SRRP_100-23_, as also reported for the pneumonococcal SecA2/Y2 system (Jiang et al. 2017). Here the putative GtfD_100-23_ and GtfE_100-23_ encoded genes share a similar organization with a GT4 in the N-terminus and a DUF1792 in the C-terminus. In addition, GtfF1_100-23_ and GtfF2_100-23_ may be part of the same gene separated by a gene encoding a putative transposase, with GtfF1_100-23_ encoding a GT4 domain in the N-terminus and part of a DUF1792 domain in the C-terminus and GtfF2_100-23_ encoding the remaining part of the DUF1792 domain. Glycosyltransferases possessing a DUF1792 has been shown to be involved in the third glycosylation step of the SRRPs, Fap1 and PsrP, from *S. parasanguinis* FW213 and *S. pneumoniae* TIGR4, respectively (Zhang et al. 2014; Jiang et al. 2017). While DUF1792 has been shown to expand the Fap1 glycan with Glc moieties in *S. parasanguinis* (Zhang et al. 2014), DUF1792 from *S. pneumoniae* showed a relaxed specificity transferring either Glc or Gal to SRR1 in *E. coli* (Jiang et al. 2017). As all additional GTs in the *L. reuteri* 100-23C SecA2/Y2 cluster contain such a domain, it is possible that only one of these enzymes is active or that there is redundancy in their function. Taken together with the SRRP_100-23_ enzymatic deglycosylation data, it is likely that SRRP_100-23_ is modified by Glc-Glc-GlcNAc or Gal-Glc-GlcNAc. Interestingly, the Glc-GlcNAc intermediate could not be identified by MALDI-ToF analysis, suggesting that the addition of the third monosaccharide onto the expanding glycan is a rapid reaction, as observed for Fap1 in *S. parasanguinis* FW213 (Zhang et al. 2014).

To date, all characterized GtfCs have been shown to add a Glc residue onto the GlcNAc core, therefore the glycosylation of SRRP_53608_ by di-GlcNAc was unexpected. The specificity of *L. reuteri* GtfC_53608_ was further supported by DSF and STD NMR analyses, showing a preference for UDP-GlcNAc, in line with the MS/GC–MS analyses. This is therefore the first report of a GtfC from the SecA2/Y2 system showing ligand specificity to UDP-GlcNAc. In addition, we showed that GtfC_53608_ (and GtfC_100-23_ to a lesser extent) bound to divalent ions, suggesting that they may contribute to optimum enzyme activity. Although these enzymes do not possess the DxD motif, commonly involved in ion binding, they harbor a DxE motif that could have a similar role. Such dependency for divalent ions is well established in Leloir GTs, and some examples have recently been reported in prokaryotic systems such as the dGT1-mediated glycosylation of Fap1 in *S. parasanguinis* (Zhang et al. 2014). However, no divalent ions have been identified so far in GtfCs from other microorganisms (Zhu et al. 2011).

SRRP_53608_ glycosylation was further confirmed by the introduction of GtfA/B/C_53608_ into *E. coli*, resulting in glycosylation of a co-expressed SRR1 domain by mono- and di-GlcNAc, as shown by MS and NMR. Heterogeneity in the glycosylation of SRRPs has been reported in SRR glycoproteins from *Streptococcus* species (Chaze et al. 2014; Zhang et al. 2014; Couvigny et al. 2017; Jiang et al. 2017), where deposition of GlcNAc moieties is not followed by further elongation of the glycan, suggesting this is a common feature among SRRPs. This heterogeneity was also observed in the glycosylation of SRRP_100-23_ (see Results section) and could explain the recognition of SRRP_100-23_ by WGA.

The NMR analysis also indicated that SRRP_53608_ is glycosylated with GlcNAcβ(1→6)-GlcNAcα moieties, providing a unique example of SRRP glycans extended with GlcNAc residues in the second position. Although only so far reported for GlcNAc residues that are directly attached onto the protein backbone, it is possible that SRRP_53608_ contains additional *O*-acetyl group moieties as previously identified in SRRPs from *S. gordonii* M99 (Seepersaud et al. 2017), *S. agalactiae* H36b (Chaze et al. 2014) and *S. salivarius* JIM8777 (Couvigny et al. 2017). In these *Streptoccocus* SRRPs, Asp2 was found to be responsible for this modification, probably on the O-6 position (Seepersaud et al. 2017). Since *L. reuteri* SecA2/Y2 clusters harbor a gene encoding a predicted Asp2 with conserved catalytic residues, Asp2 may also carry out this function in *L. reuteri*. However, since the *O*-AcGlcNAc modification is lost under the conditions used in our MALDI-ToF or GC–MS analyses (the high pH used for the release of the glycans leads to base-catalyzed ester hydrolysis and thus loss of the modification), more work is required to establish whether Asp2 functions as an acetyltransferase that modifies GlcNAc moieties of SRRP_53608_. The α-linked configuration we demonstrated here for the first time for an SRRP is in agreement with the retaining mechanism reported for GtfA from *S. gordonii* (Chen et al. 2016) and *S. pneumoniae* (Shi et al. 2014).

Interestingly, a small fraction of the gSRR1 glycans consisted of Hex-HexNAc moieties, a modification that was not found on the native protein. This suggests that GtfC could mediate the transfer of either Glc or GlcNAc in the *E. coli* glycosylation model, while showing a preference for GlcNAc in *L. reuteri* ATCC 53608, in agreement with the enzyme donor specificity and the increased levels of UDP-GlcNAc in *L. reuteri* ATCC 53608.

In *L. reuteri* 100-23C, the *Δasp2* and *ΔgtfB* mutants lost the WGA band corresponding to SRRP_100-23_, indicating that, in this strain, Asp2 and GtfB are essential for glycosylation and/or export of SRRP_100-23_. In *S. gordonii*, Asp2 is involved in both the post-translational modification and transport of SRR glycoproteins during their biogenesis (Yen et al. 2011; Seepersaud et al. 2012; Seepersaud et al. 2017). This requirement for the coupling of glycosylation and secretion has been proposed as a mechanism underpinning the co-evolution of SRR glycoproteins with their dedicated accessory SecA2/Y2 system such that the adhesin is optimally modified for binding (Seepersaud et al. 2012).

In conclusion, we showed that *Lr*SRRP adhesins are differentially glycosylated in *L. reuteri* strains 100-23C and ATCC 53608, reflecting differences in the organization of the SecA2/Y2 accessory cluster of these strains. In addition, *Lr*SRRPs from pig and rodent strains differ with respect to the number of repeat motifs and their sequences of their SRR regions (Sequeira et al. 2018). The glycosylation of SRRPs in *Lactobacillus* species, as demonstrated for the first time in this study, is likely to impact on the adhesion capacity of these strains. A recent analysis of all available genomes of *L. reuteri* strains showed that homologs of functional SRRPs (and the corresponding linked SecA2/Y2 gene cluster) were exclusively found in rodent and pig isolates, with the exception of one chicken isolate (Sequeira et al. 2018). Differences in *Lr*SRRP glycosylation profile may therefore contribute to the mechanisms underpinning *L. reuteri* adaptation to these hosts. In addition, bioinformatics analyses revealed the presence of complete SecA2/Y2 clusters with an intact SRRP in the genomes of other *Lactobacillus* species including strains from *Lactobacillus oris*, *Lactobacillus salivarius*, *Lactobacillus johnsonii* and *Lactobacillus fructivorans* (Latousakis and Juge 2018; Sequeira et al. 2018), suggesting a common role of SRR glycoproteins in adhesion to host epithelia, which may be related to the ecological context of these strains (see Duar et al. (2017) for a review). This aspect can be particularly important in the selection of probiotics targeting different vertebrate hosts. Furthermore, knowledge of the cellular pathways of glycosylation in gut symbionts expands the range of glycoengineering applications for the recombinant production of glycoprotein conjugates in different cell types.

## Materials and Methods

### Materials, strains and culture conditions

Uridine diphosphate (UDP), UDP-glucuronic acid (UDP-GlcA), UDP-*N*-acetylglucosamine (UDP-GlcNAc), UDP-*N*-acetylgalactosamine (UDP-GalNAc), UDP-glucose (UDP-Glc), UDP-galactopyranose (UDP-Gal), thymidine diphosphate (TDP)-Glc and all chemical reagents were from Merck (Gottingen, Germany), unless stated otherwise. TDP-rhamnose (TDP-Rha) was prepared as described (Wagstaff et al., 2018). Polyclonal antiserum against immobilized metal affinity chromatography (IMAC)-purified His6-SRRP_53608_-BR was raised in rabbits by BioGenes GmbH (Berlin, Germany) and provided at a titer of >1:200,000, as previously reported (Sequeira et al. 2018). The lectins used in this study were purchased from Vector Laboratories (Peterborough, UK) and are listed in Table S1.

The bacterial strains and plasmids used in this study are described in Table S2. The deMan-Rogosa-Sharpe (MRS; Oxoid, Loughborough, UK) or lactobacillus defined medium-II (LDM-II (Kotarski and Savage 1979)) medium was used for growth of *L. reuteri* strains at 37°C, and the media were supplemented with erythromycin (10 μg/mL) for *L. reuteri* 100-23C mutants. The Luria-Bertani (LB) or terrific broth-based auto induction media supplemented with trace elements (AIM; Formedium, Hunstanton, UK) were used for *E. coli* growth at 37°C, 250 rpm. The media were supplemented with the relevant antibiotics as described in Table S2.

### Lectin screening by western blot

*L. reuteri* strains were grown in LDM-II overnight at 37°C under static conditions. This culture was used to inoculate fresh LDM-II at 0.2% vol/vol. Following incubation under static conditions at 37°C overnight, the cultures were centrifuged at 4000 × *g* for 5 min and the spent media (SM) concentrated 10-fold by spin filtration using 10 kDa MWCO spin filters. The SM proteins were analyzed by SDS-PAGE, using Bis-Tris 4–12% or Tris-Acetate 3–8% NuPAGE gels (ThermoFisher Scientific, Loughborough, UK) in 3-Morpholinopropane-1-sulfonic acid (MOPS) or Tris-Acetate NOVEX buffer for 50 min at 200 V. The gels were then stained with InstantBlue protein stain (Expedeon, Over, UK). Alternatively, proteins were transferred onto PVDF membranes in NuPAGE transfer buffer, using an X-cell II blot module (ThermoFischer Scientific, Loughborough, UK) at 30 V for 2 h. The membrane was then blocked for 1 h at RT and probed with either fluorescein (*f*)-labeled lectins at 5 μg/mL or with anti-SRRP-BR_53608_ primary antibody (1000-fold dilution). Alkaline phosphatase-conjugated anti-rabbit IgG antibody Merck (Gottingen, Germany) was used as secondary antibody. Three washes with PBS supplemented with 0.1% vol/vol Tween-20 were included between antibody incubations. Bound antibody was detected using alkaline phosphatase substrate (nitroblue tetrazolium 0.1 mM, 5-bromo-4-chloro-indolyl phosphate p-toluidine 1 mM, in Tris-HCl 0.1 M containing 4 mM MgCl_2_) at pH 9.6 and scanned in a GS-800 calibrated densitometer (Bio-Rad, UK).

### *Lr*SRRP purification

*L. reuteri* 100-23C and ATCC 53608 strains were grown in LDM-II for 24 h at 37°C. The bacteria were removed following centrifugation at 10,000×*g* for 10 min. Ammonium sulfate was added to the SM at a final concentration of 60% (w/v) to precipitate the proteins. The suspension was stirred overnight. The precipitated proteins were recovered by centrifugation at 10,000 × *g* for 20 min. The proteins were resuspended in HEPES buffer (HEPES 10 mM, NaCl 150 mM, pH 7.5) and *Lr*SRRP purified by gravity flow affinity chromatography, using agarose-bound wheat germ agglutinin (agWGA). Loosely bound proteins were removed with 10 column vol of HEPES buffer and the bound proteins were eluted with six column vol of HEPES buffer containing 0.5 mM GlcNAc. The proteins were extensively dialyzed in 50 mM ammonium bicarbonate to remove free GlcNAc.

### Proteomics

Protein bands of interest were excised from SDS-NuPAGE gels and cut up to small cubic pieces. After two washes with 200 μL of ABC buffer (200 mM aqueous ammonium bicarbonate in 50% acetonitrile; ACN) for 15 min and then ACN for 10 min, the gel plugs were air-dried for 15 min. Proteins were reduced in a DL-dithiothreitol solution (200 μL, 10 mM in 50 mM ammonium bicarbonate) at 60°C for 30 min and carboxymethylated with iodoacetamide (10 mM in 50 mM ammonium bicarbonate) in the dark for an additional 30 min. The iodoacetamide solution was removed and the washing and drying steps were repeated. Trypsin Gold (10 μL; 10 ng/μL; Promega, UK) was added to the gel plugs along with equal amount of 10 mM ammonium bicarbonate. After incubation at 37°C for 3 h, 20 μL of 1% formic acid was added and the samples were further incubated at room temperature for 10 min. The solution was then transferred to a clean tube and tryptic peptides were further extracted from the gel plugs by addition of 40 μL of 50% ACN and incubation for 10 min at room temperature. The samples were pooled together and dried on a centrifugal evaporator. The peptide mixtures were analyzed by nano-scale liquid chromatographic tandem mass spectrometry (nLC MS/MS), using an Orbitrap Fusion trihybrid mass spectrometer coupled with a nano flow ultra-high performance liquid chromatography (UHPLC) system (ThermoFischer Scientific, UK). The peptides were separated on a C18 pre-column, using a gradient of 3–40% ACN in 0.1% formic acid (vol/vol) over 50 min at a flow rate of 300 nL/min at 40°C. The peptides were fragmented in the linear ion trap by a data-dependent acquisition method, selecting the 40 most intense ions. Mascot (Matrix Science, UK) was used to analyze the raw data against an in-house maintained database of the *L. reuteri* and/or *E. coli* proteome. The tolerance on parent ions was 5 ppm and on fragments was 0.5 Da. Carboxymethylation of cysteine was selected as fixed modification and oxidation of methionine as variable modification. One miscleavage was allowed.

### Enzymatic treatment of SRRPs

*Lr*SRRP was treated with α-glucosidase from *Saccharomyces cerevisiae*, α-galactosidase from green coffee beans, β-glucosidase from almonds or β-galactosidase from *Αspergillus oryzae* (0.5 U/μL; Merck Gottingen, Germany) in 50 mM sodium acetate, 5 mM CaCl_2_, pH 6 for 16 h. The reaction products were analyzed by SDS-PAGE and western blot, as described above.

### Glycan analysis by Matrix Assisted Laser Desorption/ionization Time of Flight Mass Spectrometry (MALDI-ToF)

*Lr*SRRP glycans were released by β-elimination, after treatment of the purified proteins with 1 M NaBH_4_ in 50 mM NaOH for 16 h at 45°C. Excess of NaBH_4_ was neutralized by the addition of acetic acid, before sodium ions were removed by ion-exchange chromatography, using a DOWEX 50Wx8 H^+^ column. Glycans were collected in the flow-through and wash fractions using 5% acetic acid. These fractions were pooled and freeze-dried, prior to permethylation of the glycans with 300 μL NaOH – anhydrous dimethylsulfoxide (DMSO) slurry and 400 μL iodomethane. The reaction was incubated at room temperature for 60 min under vigorous shaking and quenched by the dropwise addition of H_2_O, until fizzing stopped. The permethylated glycans were extracted in 2 mL chloroform, washed three times with 2 mL H_2_O. After drying the organic phase under nitrogen, glycans were dissolved in 50 μL aqueous methanol 50% vol/vol and loaded onto a pre-washed with methanol, acetonitrile and water Empore™ C18-SD cartridge (7 mm; Merck, Germany). Hydrophilic contaminants were washed with 500 μL H_2_O and 400 μL 15% vol/vol aqueous acetonitrile. Permethylated carbohydrates were eluted with 400 μL of 35%, 50% and 75% vol/vol aqueous ACN. The eluants were dried under a gentle stream of nitrogen, dissolved in 10 μL of TA30 [30% (vol/vol) ACN, 0.1% (vol/vol) trifluoroacetic acid] and mixed with equal amount of 2,5-dihydroxybenzoic acid (DHB; Sigma-Aldrich, UK; 20 mg/mL in TA30), before being spotted onto an MTP 384 polished steel target plate (Bruker, UK). The samples were analyzed by MALDI-ToF, using the Bruker Autoflex™ analyzer mass spectrometer (Bruker, UK) in the positive-ion and reflectron mode.

### Monosaccharide analysis by gas chromatography (GC)–MS

*Lr*SRRPs were treated with methanolic HCl (1 M) for 16 h and 5 μg of myo-inositol added as internal standard. Silver carbonate (~50 mg) was added to the solution, followed by 100 μL acetic anhydride and the reactions were incubated at room temperature for 16 h in the dark. Lipids were removed by three washes with heptane and the remaining methanolic phase was dried under a gentle nitrogen flow. Tri-Sil HTP reagent (200 μL) (ThermoFischer Scientific, Loughborough, UK) was added to the dried sample and the reaction was incubated at 80°C for 30 min. The solution was dried under nitrogen and 1 mL of hexane was used to extract sugars by sonication for 15 min. The samples were transferred to clean vials, dried and dissolved in dichloromethane (100 μL) before injection onto the GC–MS. The samples were analyzed on an Agilent 7890B GC–MS system paired with an Agilent 5977 A mass spectrometry detector (Agilent, UK), using a BPX70 column (SGE Analytical Science, Australia). Helium was used as the carrier gas. The inlet was maintained at 220°C, 12.9 psi, and 23 mL/min flow. The injection volume was 1 μL in split mode (1:20). The oven temperature increased initially from 100°C to 120°C over 5 min, followed by a second increase from 120°C to 230°C over 40 min.

### Cloning, expression and purification of glycosyltransferases

For the production of recombinant GtfC_53608_, the coding region of *gtfC*_53608_ was amplified by PCR from the genomic DNA of *L. reuteri* ATCC 53608 using 0907-F and 0907-R primers (Table S2) and cloned into a pOPINF vector linearized with *Kpn*I-HF and *Hin*dIII-HF, using the In-Fusion HD kit (Clonetech, California, USA), following the manufacturer’s instructions. The recombinant vector was used to transform *E. coli* BL21 (DE3). AIM medium was inoculated with an overnight culture of the recombinant clone at 1%. The fresh culture was incubated at 37°C for 3 h and then 16°C for 48 h. The cells were harvested by centrifugation at 10,000×*g*, resuspended in Tris buffer (Tris-HCl 50 mM, NaCl 150 mM, pH 7.5). The bacteria were lysed by 10 cycles of sonication and soluble, His_6_-tagged proteins were purified by immobilized metal ion affinity chromatography (IMAC). Bound proteins were eluted with Tris buffer containing 100 mM EDTA, concentrated by spin filtration, using a 10 kDa MWCO Vivaspin® Turbo 15 spin filter (Sartorious, Gottingen, Germany) and buffer-exchanged in Tris buffer using PD10 desalting columns (GE Healthcare Lifesciences, Little Chalfont, UK), following the manufacturer’s instructions. Purified recombinant GtfC_100-23_ produced in *E. coli* was a kind gift from Carl Young (Prozomix, UK).

### Glycosylation of SRR1

For the glycosylation of SRR acceptor in *E. coli*, an artificial *gtfCAB*_53608_ operon was cloned into pETcoco™-1 (Merck, Gottingen, Germany). Briefly, primer pairs nss_F and nss_R or gtfA_F and gtfB_R (Table S2) were used together with ATCC 53608 template DNA to generate two PCR products of 1055 bp or 2905 bp, respectively. Next, equimolar amounts of these products were mixed and used as template together with the primers nss_F and gtf_R (Table S2) to generate the final 3915 bp splice PCR product. Subsequently, the *Not*I restricted product was cloned into pETcoco™-1 that had been restricted with *Sph*I, treated with T4-polymerase (New England Biolabs) and subsequently cut with *Not*I, resulting in pETcoco_*gtfCAB*_53608_. Partial *srr* gene was cloned into pET-15b. Briefly, a primer pair dsrr_F and dsrr_R (Table S2) was used to amplify a 487 bp product encoding the 81-236 aa region of SRRP_53608_ that corresponds to the first serine-rich repeat region (SRR1) of SRRP_53608_. Restriction sites incorporated into the primers (Table S2) enabled the restriction with *Nde*I and *Bam*HI and the subsequent ligation into pET-15b that had been restricted in the same way resulting in pET-15b_*srr*1. Both pETcoco_*gtfCAB*_53608_ and pET-15b_*srr*1 were then used to transform *E. coli* BL21 (DE3). Induction of the expression and purification of the His-tagged SRR1 were performed as described above for GtfC_53608_.

### Differential scanning fluorimetry (DSF)

DSF was used to assess glycosyltransferase – sugar donor interactions by measuring changes in the melting temperature (Tm) of the protein upon interaction with sugar nucleotides. The reactions were set up at a final volume of 20 μL in Tris-HCl 50 mM, pH 7.5. Proteins were used at a final concentration of 10 μM and SYPRO Orange (ThermoFischer Scientific, UK), the fluorescent dye used in the assay was used at 5× final concentration. Ligand and ion concentration ranged from 0 to 50 mM. To measure the effect of divalent ions on the protein–ligand interaction, sugar donors were used at 4 mM and divalent ions at 5 mM. The reactions were initially kept at 10°C for 10 min and then the temperature increased in a step-wise manner, with increments of 0.5°C every 15 s, up to 90°C. Measurement of the fluorescence was taken every 15 s on a Real-Time PCR Detection System (Bio-Rad CFX96 Touch™). The results were analyzed using CFX Manager 3.5 (Bio-Rad, UK).

### Saturation Transfer Difference (STD) NMR experiments

Proteins were exchanged using an Amicon centrifuge filter unit with a 3 kDa MW cutoff in 20 mM *d*_19_-2,2-bis(hydroxymethyl)-2,2′,2″-nitrilotriethanol pH 7.4 (uncorrected for the deuterium isotope effect on the pH glass electrode) and 50 mM NaCl. Ligands (UDP, UDP-GlcNAc, UDP-Glc, UDP-Gal) were dissolved in 20 mM *d*_19_-2,2-bis(hydroxymethyl)-2,2′,2″-nitrilotriethanol pH 7.4, 50 mM NaCl. The final ligand concentration was measured using 4,4-dimethyl-4-silapentane-1-sulfonic acid as an internal standard of known concentration. The protein concentration in the NMR tube (volume 500 μL) was 28 μM for GtfC_100-23_ and 21 μM for GtfC_53608_. Ligands were used in concentrations ranging from 0.3 to 3.5 mM. The STD NMR spectra were performed on a Bruker Avance 500 MHz at 298 K following published methodology (Mayer and Meyer 1999). The on- and off-resonance spectra were acquired using a train of 50 ms Gaussian selective saturation pulses at a fixed saturation time of 2 s (for *K*_D_ determination) or variable saturation time from 0.5 s to 4 s (for binding epitope mapping determination). The water signal was suppressed by using the WATERGATE technique as described in Piotto et al. (1992) while the remaining protein resonances were filtered using a T_2_ filter of 40 ms. The selective on-resonance irradiation was performed at 0.7 ppm while the off-resonance irradiation was performed at 40 ppm. The spectra were performed with a spectral width of 5 KHz and 32768 data points. For determination of apparent *K*_D_, the spectra were collected with either 32 or 64 scans and 8 dummy scans at 2 s saturation time, while for the binding epitope mapping the spectra were collected with 512 scans, 8 dummy scans and a 4 s relaxation delay for all the spectra. For each ligand interacting with GtfC_100-23_ or GtfC_53608_, the STD build up curve was obtained and the STD_0_ parameter (STD factor at time 0) was used to derive the binding epitope. STD_0_ was obtained by fitting the build-up curve data to the equation STD(*t*_sat_) = STD_max_ * (1–exp(–*k*_sat_ * *t*_sat_)) where the STD_0_ factor is calculated by STD_max_ * *k*_sat_ = STD_0_. For each proton STD_0_ factors were normalized to the highest STD_0_ within each ligand, and expressed as relative STD_0_(%) so that the binding epitope mappings could be derived.

### Sugar nucleotide profiling by liquid chromatography coupled with tandem mass spectrometry (LC–MS/MS)

*L. reuteri* strains 100-23C and ATCC 53608 were grown in 1 l MRS until OD_600_ reached ~1.0, harvested by centrifugation at 10,000×*g* for 10 min, washed three times in ice-cold PBS, and resuspended in 70% ethanol. UDP-GlcA (1.6 nmol/g wet pellet) was added to the suspension as an internal standard. Cells were then lysed for five cycles of 50 s each using 100 μm long glass beads on a FastPrep®-24 homogenizer (MP Biomedicals, UK). Cells were kept on ice for 2 min between cycles. After centrifugation at 10,000 ×*g* for 20 min, the supernatant was recovered and ethanol was evaporated under a stream of nitrogen. The aqueous residue was freeze-dried and contaminating lipids were extracted with butan-1-ol as previously described (Turnock and Ferguson 2007). Sugar nucleotides were dissolved in ammonium bicarbonate 5 mM and extracted using ENVI-Carb cartridges as described in Rabina et al. (2001). The samples were dissolved in 50 μL formic acid (80 mM) brought to pH 9.0 with ammonia (mobile phase A) and analyzed on a surface-conditioned porous graphitic carbon (PGC) column (Hypercarb™, 100 × 1 mm, 5 μm; ThermoFischer. Loughborough, UK) with detection by tandem quadrupole mass spectrometer in electrospray ionization mode (ESI-MS/MS) (Pabst et al. 2010), using Xevo TQ-S coupled to an Acquity UPLC (Waters, Elstree, UK), as described previously (Rejzek et al. 2017). Available sugar nucleotide standards (10 μM) were injected (5 μL) to determine retention times. The mass spectrometer was operated in multiple reaction monitoring (MRM) mode. MRM transitions for sugar nucleotide standards were generated using IntelliStart software as described in Rejzek et al. (2017). For generic groups (e.g., UDP-*N*-acetylhexosamines, UDP-HexNAc) or where authentic standard was not available (UDP-*N*-acetylmuramic acid, UDP-MurNAc) predicted MRM functions were generated (Turnock and Ferguson 2007) (Supplementary Table S1). MassLynx software (Waters) was used to collect, to analyze and to process data. When needed, co-injection of samples with standards was used to further confirm analyte identification. Analysis of three biological replicates was performed. To ensure reproducible retention times, the Hypercarb PGC column was freshly regenerated before the analysis, as described in Supplemental methods.

## Supplementary Material

Supplementary Data

## Abbreviations

AST, alanine-serine-threonine; BR, binding region; DSF, differential scanning fluorimetry; GT, glycosyltransferase; IMAC, immobilized metal affinity chromatography; SM, spent media; SRRP, serine-rich repeat protein; STD, saturation transfer difference; UDP, uridine diphosphate.
