# Supplementary material for: Serine-rich repeat protein adhesins from Lactobacillus reuteri display strain specific glycosylation profiles
Source: Glycobiology. 2018 Nov 23;29(1):45–58. doi: 10.1093/glycob/cwy100 (PMC6291802; doi:10.1093/glycob/cwy100)
Supplement: Supplementary Data [file cwy100_revised_srr_glycobiology_supplementary_data.docx]

**Supporting tables**

**Table S1.** Retention times, MRM transitions and quantification of the most abundant sugar nucleotides detected in *L. reuteri* 100-23C and ATCC 53608 strains.

| Sugar Nucleotide | MRM  transitions | Fragment | Retention time:  relative  (average ± SEM* [min]) | Concentration in 100-23  (nmol/g of wet pellet) | Concentration in ATCC 53608  (nmol/g of wet pellet) |
| --- | --- | --- | --- | --- | --- |
| UDP-α-d-glucose  (UDP-Glc) | 565 → 323  565 → 79 | [NMP-H]^-^  [H_3_PO_4_-H_3_O]^-^ | 1.00  (11.72±0.04)** | 230.72±73.06 | 884.71±115.76 |
| UDP-α-d-galactopyranose  (UDP-Gal) | 565 → 323  565 → 159 | [NMP-H]^-^  [H_4_P_2_O_7_-H_3_O]^-^ | 0.93  (10.92±0.08)** | 1.59±0.26 | 155.21±33.32 |
| UDP-*N*-acetyl-α-d-glucosamine  (UDP-GlcNAc) | 606 → 385  606 → 159 | [NDP-H_3_O]^-^  [H_4_P_2_O_7_-H_3_O]^-^ | 1.03  (12.03±0.04)** | 147.94±15.55 | 1322.73±175.26 |
| UDP-*N*-acetylmuramic acid  (UDP-MurNAc) | 678 → 403  678 → 323 | [NDP-H]^-^  [NMP-H]^-^ | 1.11  (13.01±0.05) | 54.18±3.32 | 148.40±13.46 |
| TDP-β-l-rhamnose  (TDP-Rha) | 547 → 321  547 → 225 | [NMP-H]^-^  c[Rha-1-P-H_3_O]^-^ | 1.48  (17.36±0.05)** | 449.61±48.52 | 14.05±3.93 |
| 5''-(adenosine 5'-pyrophosphoryl)  -d-ribose (ADP-Rib) | 558 → 346  558 → 159 | [NMP-H]^-^  [H_4_P_2_O_7_-H_3_O]^-^ | 1.73  (20.28±0.06) | 459.52±243.33 | 370.86±159.74 |

*average of 3 measurements; **the identity was confirmed by co-injection with standard

**Table S2 Bacterial strains, lectins oligonucleotides and plasmids used in this study**

| **Bacterial Strains** | **Reference / supplier** | |
| --- | --- | --- |
| *L. reuteri* 100-23C wild type (WT) | (Frese, S.A., Benson, A.K., et al. 2011) | |
| *L. reuteri* 100-23 *Δsrr* (Lr_70902) | (Frese, S.A., Benson, A.K., et al. 2011) | |
| *L. reuteri* 100-23 *Δasp2* (Lr_70890) | (Frese, S.A., Benson, A.K., et al. 2011) | |
| *L. reuteri* 100-23 *ΔgtfB* (Lr_70894) | (Frese, S.A., Benson, A.K., et al. 2011) | |
| *L. reuteri* ATCC 53608 | ATCC | |
| *E. coli* DH5α | ThermoFischer Scientific | |
| *E. coli* BL21 (DE3) | New England Biolabs | |
| **Lectins** | **Specificity** | **Supplier** |
| *Fluorescein-*Concanavalin A (*f-*ConA) | α-mannose | Vector laboratories |
| *Fluorescein-Lotus tetragonolobus* (*f-*LTL) | α-L-fucose | Vector laboratories |
| *Fluorescein-*Peanut agglutinin (*f*-PNA) | Gal-(β-1,3)-GalNAc (T-antigen) | Vector laboratories |
| *Fluorescein-Ulex europaeus agglutinin* (*f*-UEA) | α-L-fucose | Vector laboratories |
| *Fluorescein-Ricinus communis* agglutinin (*f*-RCA) | Gal or GalNAc | Vector laboratories |
| *Fluorescein-*Wheat germ agglutinin (*f-*WGA) | GlcNAc or sialic acid | Vector laboratories |
| Fluorescein-*Sambucus nigra agglutinin (f-*SNA) | α-2,6-linked sialic acid | Vector laboratories |
| Agarose-Wheat germ agglutinin (agWGA) | GlcNAc or sialic acid | Vector laboratories |
| **Oligonucleotides** | **Reference** | |
| **nss_F**  ATTGTTTTATAATGGAGGGATTATCATTGAC | This study | |
| **nss_R**  CAGTCATACTTTCCTCCATCATTATTGATAATATAATTTAAATAC | This study | |
| **gtfA_F**  GTATTTAAATTATATTATCAATAATGATGGAGGAAAGTATGACTG | This study | |
| **gtfB_R**  ATGCGGCCGCTTATTCACTTTGCAAGGCTCCAATC | This study | |
| **dsrr_F**  TATCATATGGTTTCACCAACAGAAGTCCAC | This study | |
| dsrr_R  ATGGATCCTAGGTTGCTGGCAAGATGGTTGC | This study | |
| **0907-F**  AAGTTCTGTTTCAGGGCCCGTTGACAGTTCATATTACTAACCTTT | This study | |
| **0907-R**  ATGGTCTAGAAAGCTTTATTGATAATATAATTTAAATACTGCTTC | This study | |
| **Recombinant DNA (antibiotic resistance)** | **Reference / Supplier** | |
| pOPINF (carbenicillin^+^, 100μg/ml) | Addgene | |
| pETcoco-1 (chloramphenicol^+^_,_ 25 μg/ml) | Merck Millipore | |
| pET15b (carbenicillin^+^, 100μg/ml) | Merck Millipore | |
| pOPINF-*gtfA* (carbenicillin^+^, 100μg/ml) | This study | |
| pOPINF-*gtfB* (carbenicillin^+^, 100μg/ml) | This study | |
| pOPINF-*gtfC* (carbenicillin^+^, 100μg/ml) | This study | |
| pETcoco-1-*gtfCAB* (chloramphenicol^+^_,_ 25 μg/ml) | This study | |
| pET-15b-*srr*1 (carbenicillin^+^, 100μg/ml) | This study | |

**Supporting figures**


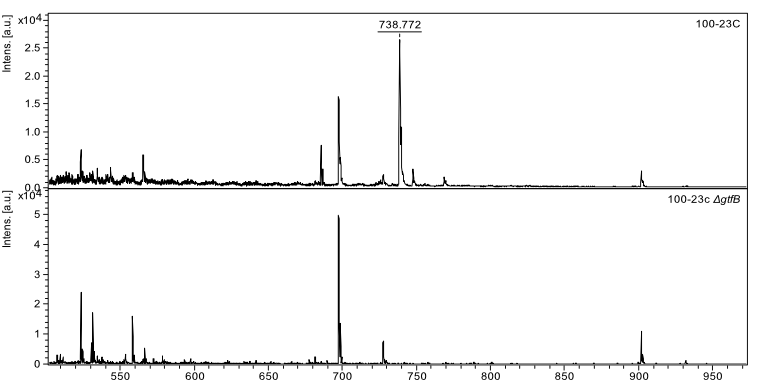


**Figure S1**. **MALDI-ToF analysis of released glycans from SM proteins from *L. reuteri* 100-23C WT and Δ*gtfB* mutant.** The results show that the peak at 738 Da is missing from the ΔgtfB mutant, suggesting that GtfB is involved in the synthesis of this glycan and therefore this glycan is found on SRRP_100-23_.


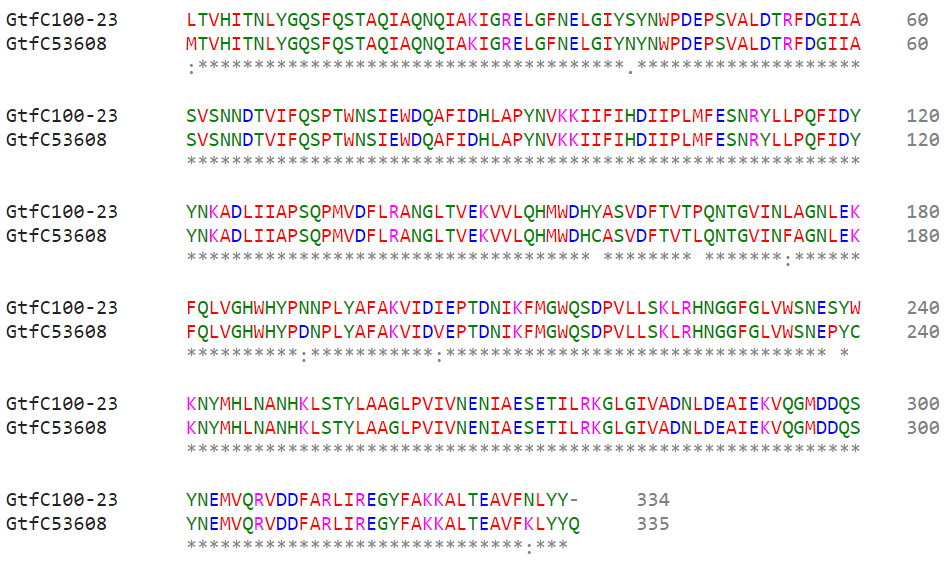


**Figure S2. Alignment of the amino acid sequence of GtfC_100-23_ and GtfC_53608._** The two sequences are 97% concenrved.


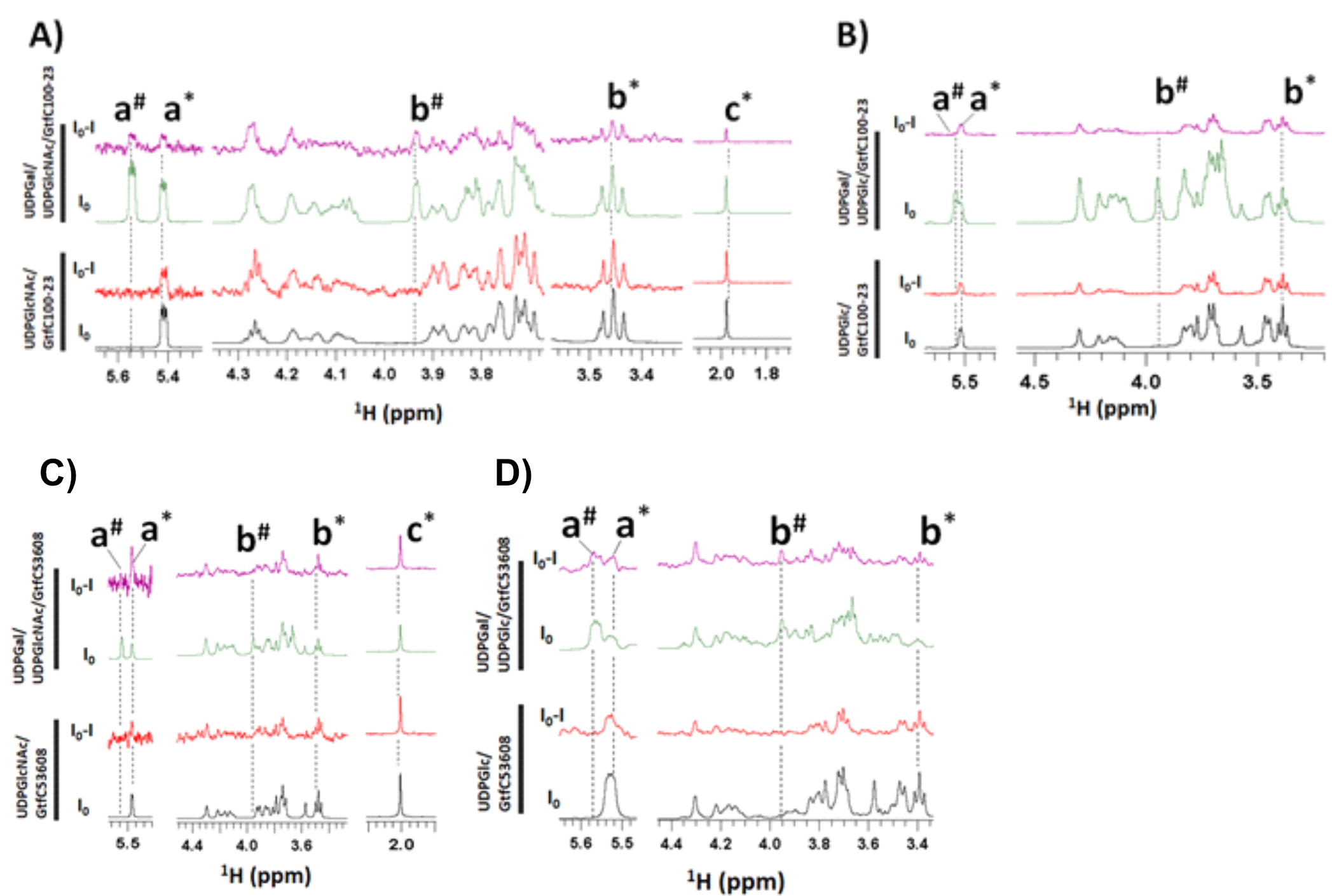


**Figure S3. STD NMR competition experiments** **of GtfC_100-23_ (top panels) and GtfC_53608_ (bottom panels) in complex with sugar nucleotides. (A)** GtfC_100-23_/UDP-GlcNAc and (**B)** GtfC_100-23_/UDP-Glc in the presence (top) and in the absence (bottom) of UDP-Gal. The concentration of UDP-Gal was 1.8 mM while the concentration of UDP-Glc or UDP-GlcNAc was 1.5 mM and the protein concentration was 28 μM. UDP-Gal displaces UDP-GlcNAc from its complex with GtfC_100-23_, but is unable to displace UDP-Glc from the GtfC_100-23_/UDP-Glc complex. In **A)** or **B)** the resonances under the # symbol belong to the galactose moiety of UDP-Gal and were monitored for binding confirmation while the resonances labeled with the * symbol belong to either UDP-GlcNAc (H1”(a*); H6”(b*); CH3 (c*)) or UDP-Glc (H1”(a*); H6”(b*)), and were used to quantify the displacement. In **A)** for the GtfC_100-23_/UDP-GlcNAc complex, reductions in STD factors of 42%, 58% and 53% for H1”(a*); H6”(b*); CH3 (c*) were observed, respectively, in the presence of UDP-Gal. The presence of STD signals of UDP-Gal confirmed binding, suggesting a similar affinity of GtfC_100-23_ for UDP-Gal and UDP-GlcNAc. In **B)** for the GtfC_100-23_/UDP-Glc complex reductions in STD factors of 16% and 11% for H1”(a*) and H6”(b*) were observed, respectively, in the presence of UDP-Gal. Although some interaction might occur, the displacement of UDP-Glc was very low, in agreement with a very low bound population of UDP-Gal (below STD NMR detection), confirming that GtfC_100-23_ shows a higher affinity for UDP-Glc. **(C)** GtfC_53608/_UDP-GlcNAc and (**D)** GtfC_53608_/UDP-Glc in the presence (top) and in the absence (bottom) of UDP-Gal. The concentration of UDP-Gal was 1.8 mM while the concentration of UDP-Glc or UDP-GlcNAc was 1.5 mM and the protein concentration was 21 uM. UDP-Gal displaces UDP-Glc from its complex with GtfC_53608_, but is unable to displace UDP-GlcNAc from the GtfC_100-23_/ UDP-GlcNAc complex. In **C)** or **D)** the resonances under the # symbol belong to the galactose moiety of UDP-Gal and were monitored for binding confirmation, while resonances labeled with the * symbol belong to either UDP-GlcNAc (H1”(a*); H6”(b*); CH3 (c*)) or UDP-Glc (H1”(a*); H6”(b*)), and were used to quantify the displacement. In **C)** for the GtfC_53608_/UDP-GlcNAc complex, reductions in STD factors of 0% 10% and 0% for H1”(a*); H6”(b*) and CH3 (c*) were observed, respectively, in the presence of UDP-Gal. Although some interaction might occur, the displacement of UDP-GlcNAc was very low, in agreement with a very low bound population of UDP-Gal (below STD NMR detection), confirming that GtfC_53608_ shows a higher affinity for UDP-GlcNAc. In **D)** for the GtfC_53608_/UDP-Glc complex, reductions in STD factors of 60% and 38% for H1”(a*) and H6”(b*) were observed, respectively, in the presence of UDP-Gal. The presence of STD signals of UDP-Gal confirmed binding and competition with UDP-Glc suggest a similar affinity of GtfC_53608_ for UDP-Gal and UDP-Glc.


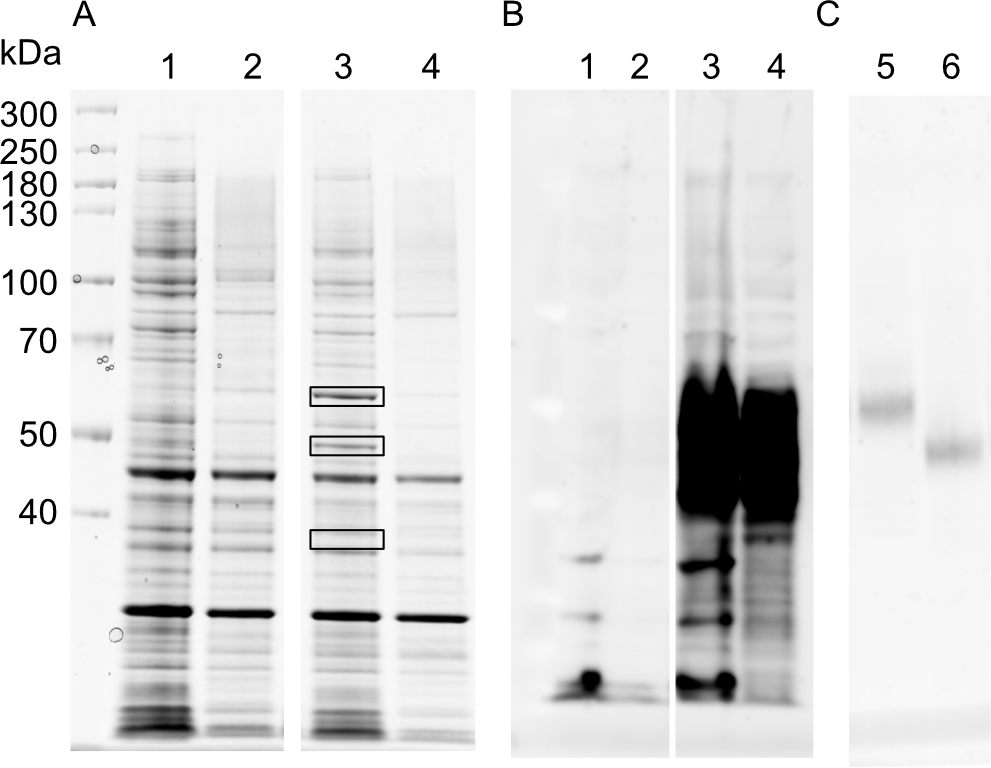


**Figure S4. Electrophoresis analysis of pET-15b_*srr*1 and *E. coli* BL21 (DE3)** **pETcoco-1_*gtfCAB*/pET-15b_*srr*1 lysates.** SDS-PAGE analysis (**A**) and western blot analysis using *f-*WGA (**B**) of *E. coli* BL21 (DE3) 1. Insoluble fraction of *E. coli* BL21 (DE3) pET-15b_*srr*1 after lysis, 2. soluble fraction of *E. coli BL21* (DE3) pET-15b_*srr*1, 3. Insoluble fraction of *E. coli* BL21 (DE3) pETcoco-1_*gtfCAB*/pET-15b_*srr*1 after lysis, 2. soluble fraction of *E. coli* BL21 (DE3) pETcoco-1_*gtfCAB*/pET-15b_*srr*1. Proteins marked in boxes were trypsinised and analysed by LC-MS. (**C**) Western blot analysis of gSRR1 before (5) and after (6) β-*N*-acetylhexosaminidase_f_ treatment, using *f*-WGA.

**
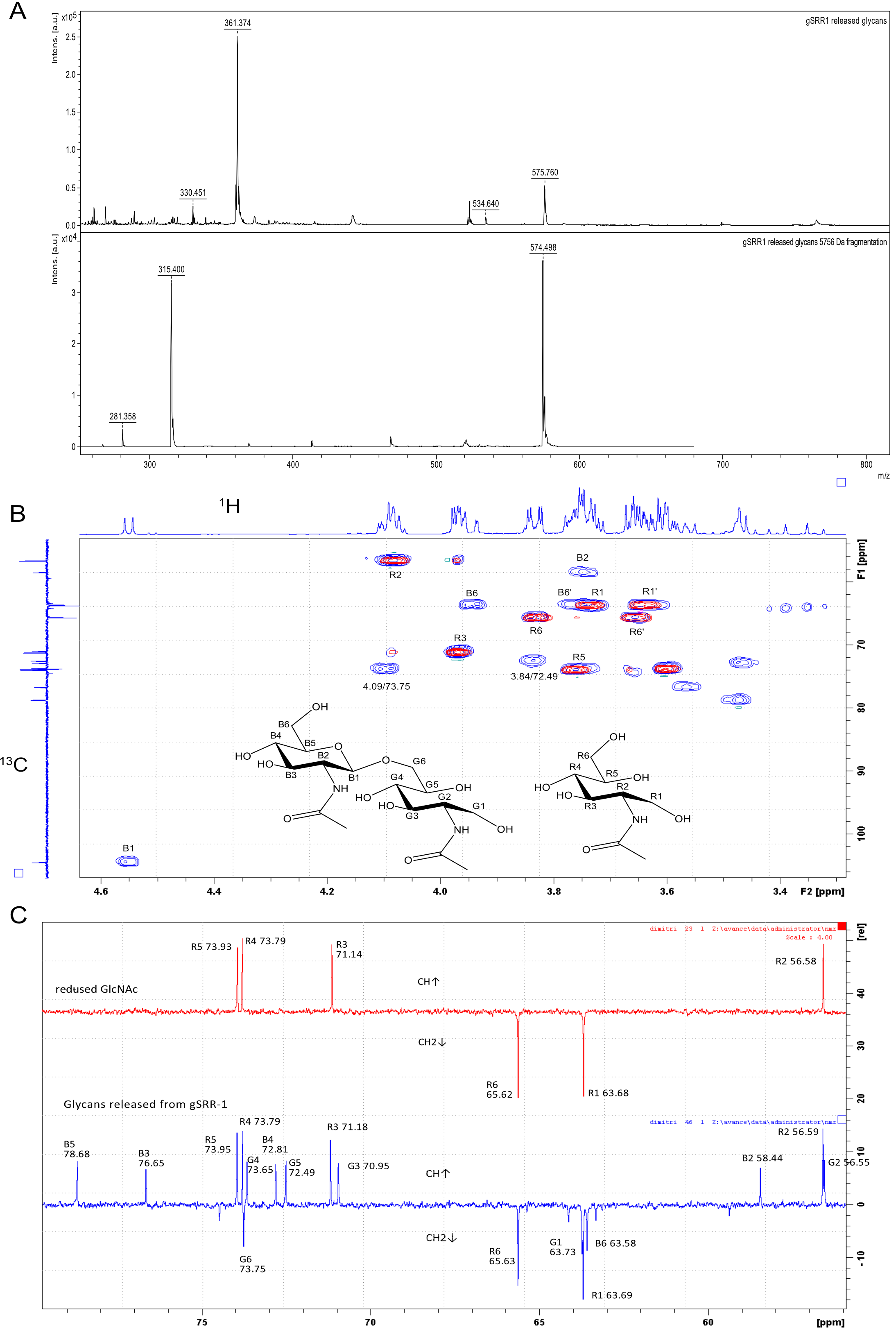
**

**Figure S5. Structural characterisation of the gSRR1-released glycans A) MALDI-ToF analysis of released glycans from recombinant gSRR1.** The results show a monosaccharide peak at 330 (HexNAc), and two disaccharide peaks at 534 Da (Hex-HexNAc) and 575 Da (di-HexNAc) (top). Fragmentation of the 575 Da peak (bottom) confirmed the structure of the disaccharide. **B)** HSQC spectrum of glycans released from gSRR1 (blue) with peaks of free GlcNAc-ol superimposed (red). The glycan fraction is a mixture of disaccharide containing β-GlcNAc (*B*) and GlcNAc-ol (*G*) and free GlcNAc-ol (*R*). Peaks were assigned by a combination of 2D and DEPTQ NMR experiments and comparison with reference standards (Table 1). The coupling constant *J*_1,2_ (8.5Hz) of the only anomeric proton *B*1 shows that the disaccharide has a β-linkage. The HMBC spectrum (not shown) had an inter-residue cross peak at 4.55/73.75 ppm (*B*1→*G_x_*) where *G_x_* is the GlcNAc-ol linkage position. **C)** Partial DEPTQ (^13^C) spectra of glycan fraction (blue) and free GlcNAc-ol (red). The ^13^C chemical shifts of the peaks labelled R in the glycan fraction are identical with those of the reference standard GlcNAc-ol. This confirmed that the fraction contained a mixture of GlcNAc-ol (R) and disaccharide (B, G) and the peak heights give a ratio of 60% (GlcNAc-ol) to 40% (disaccharide). It is likely that these figures reflect the ratio of mono-GlcNAc to di-GlcNAc side chains in the gSRR1 protein (see also **Figure S7D**). There were two unassigned cross-peaks in **Figure S6A** with δ^13^C of 73.75 (shown by HMBC to be the δ^13^C of Gx, the GlcNAc-ol linkage position in the disaccharide) and 72.49 ppm. The DEPTQ spectrum shows that δ73.75 is from a CH2 group whilst δ72.49 is a CH. Thus, Gx must be either G1 or G6, the only CH2 groups in GlcNAc-ol. The DEPTQ spectrum of the glycan fraction has some obviously identifiable pairs of closely related strong/ weak signals e.g. R2/G2, R3/G3 and (δ63.69/63.73, both CH2) which must be R1/G1 but R6 (δ65.63) has no such close neighbour. Gx, the linkage position of GlcNAc-ol in the disaccharide, is therefore G6 with the large downfield displacement from δ65.63 in free GlcNAc-ol as expected for linkage formation at the 6-position. The ^1^H/^13^C assignment of the disaccharide is completed with results from other 2D experiments such as COSY (**Table 1**). The disaccharide is identified as β-GlcNAc-(1→6)-GlcNAc-ol, derived from a β-GlcNAc-(1→6)-GlcNAc side chain. An additional very minor doublet at δ^1^H 4.50 (H1, with H2 at δ3.34) could be β-Glc, possibly associated with the Hex-HexNAc-ol identified by MALDI-ToF MS.


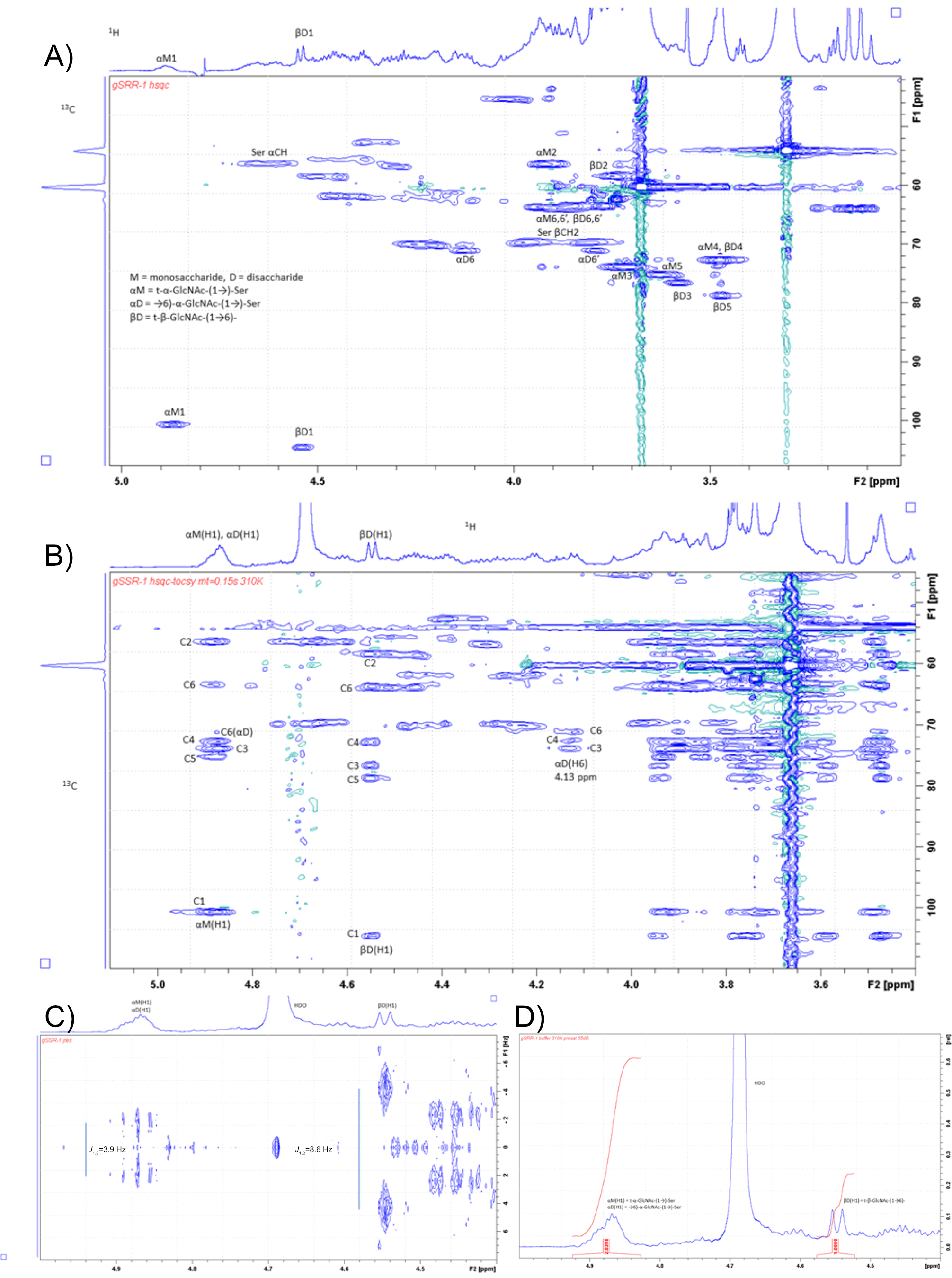


**Figure S6. NMR analysis of intact gSRR1. A)** HSQC spectrum of the protein gSRR1. The spectrum displays two anomeric peaks at δ4.87/100.61 (αM1) and δ4.54/104.51 (βD1). The ^1^H signal of βD1 is a doublet (*J*_1,2_ = 8.5Hz) and the ^1^H/^13^C signals labelled βD1-βD6 have the same chemical shifts as those labelled *B1-B6* in Figure S8. βD is therefore identified as a t-β-GlcNAc unit, the same as *B* in the disaccharide identified above. The signal labelled αM1 must belong to the sugar unit that is linked to the protein Ser residues. The αM2 HSQC signal indicates the presence of an α-linked GlcNAc unit (δ^1^H/^13^C = 3.92/56.35; compare values of δH2/C2 for α- and β-GlcNAc in the Reference standards section of **Table 1**). Conclusive evidence for the presence of α-linked GlcNAc unit(s) is provided in Figure S12 and the reason for the apparent broadening of the αM1 ^1^H signal is discussed. The HMBC spectrum of gSRR1 (not shown) had a cross peak at δ4.54/71.1. This is an inter-residue cross peak since δ^1^H = 4.54 is the chemical shift of βD1 but δ^13^C = 71.1 does not correspond to any of the ^13^C chemical shifts of the βD unit. It is shown in Figure S11 that the δ^13^C = 71.1 signal is αD6, i.e. C6 of the 6-linked α-GlcNAc unit in the disaccharide side-chain. **B)** HSQC-TOCSY spectrum of gSRR1 protein. In this display all the ^13^C chemical shifts for each unit may be read off the ^13^C axis using the relevant vertical columns located at αM(H1) and βD(H1) on the ^1^H axis. Similarly, the signals in the horizontal rows originating at αM(C1) and βD(C1) allow all the ^1^H chemical shifts within each unit to be read off the ^1^H axis. Each ^1^H chemical shift is associated with its own column in which the ^13^C chemical shifts of the relevant unit are repeated. The ^13^C chemical shifts for unit βD are in accord with those determined for the disaccharide t-β-GlcNAc and listed in **Table 1**. We have also noted that the δH2/C2 values for unit αM are suggestive of an α-linked GlcNAc. The pattern of the other ^13^C chemical shifts seen in the αM column supports this. Furthermore, since only δC1 differs greatly from the δ^13^C values listed in **Table 1** for the standard α-GlcNAc it is apparent that αM is a t-α-GlcNAc (δC1 is altered by the linkage formation to Ser). In particular, δC6 = 63.44 which is a value typical of a free -CH_2_OH group but not of a 6-linked sugar unit. Therefore, it appears that a proportion of the α-GlcNAc units linked via Ser to the protein are present as single residues. The same αM column however also carries a weak signal labelled C6(αD) at δ^13^C = 71.1 ppm. This was the δ^13^C of the inter-residue cross peak found in the HMBC spectrum correlated to βD(H1) at δ4.54 and is the chemical shift of the carbon involved in the glycosidic linkage with βD. δ^13^C = 71.1 is a C6 as it is correlated to two ^1^H chemical shifts (4.13, 3.80 ppm – labelled αD6, αD6’ in **Figure S7A**). Cross peaks were observed in the NOESY spectrum of gSRR1 (not shown) at δH4.55/(4.13, 3.80) corresponding to βD(H1)→αD(H6, H6’) and confirming the proposed linkage. Several ^13^C signals (C6, C4, C3) associated with the same unit are seen in the HSQC-TOCSY at δ^1^H = 4.13 ppm. These ^13^C chemical shifts and the similarity of δH1 to that of αM all indicate that the unit in question is a 6-linked α-GlcNAc (αD), and that it is this unit of the disaccharide, β-GlcNAc-(1→6)-α-GlcNAc, that is α-linked via Ser to the protein. **C)** J-resolved spectrum of gSRR1 protein (anomeric region). This spectrum shows that whereas the t-β-GlcNAc of the disaccharide has a single well resolved doublet anomeric signal (*J*_1,2_ = 8.5 Hz), the α-GlcNAc anomeric signal consists of a series of doublets (all with *J*_1,2_ = 3.9 Hz in agreement with the α-configuration) over a range of chemical shifts from 4.91-4.85 ppm. This can be understood since the α-GlcNAc units are all linked to Ser residues in slightly different environments because of the protein secondary structure: this in turn will influence the α-GlcNAc δH1 values. **D)** ^1^H spectrum and integration of gSRR1 protein (anomeric region). The integration ratio is 2.8:1 (αH1:βH1). Since 1 unit of αH1 must be attributed to α-GlcNAc that is part of the disaccharide, the ratio of mono-α-GlcNAc side chain: disaccharide side chain is 1.8:1 i.e. 64% mono- to 36% disaccharide in fair agreement with the ratio of GlcNAc-ol: disaccharide found above (**Figure S7**).
